# In silico prioritisation of candidate genes for prokaryotic gene function discovery: an application of phylogenetic profiles

**DOI:** 10.1186/1471-2105-10-86

**Published:** 2009-03-17

**Authors:** Frank PY Lin, Enrico Coiera, Ruiting Lan, Vitali Sintchenko

**Affiliations:** 1 Centre for Health Informatics, University of New South Wales, Sydney, Australia; 2 School of Biotechnology and Biomolecular Sciences, University of New South Wales, Sydney, Australia; 3 Centre for Infectious Diseases and Microbiology, Western Clinical School, University of Sydney, Sydney, Australia

## Abstract

**Background:**

*In silico *candidate gene prioritisation (CGP) aids the discovery of gene functions by ranking genes according to an objective relevance score. While several CGP methods have been described for identifying human disease genes, corresponding methods for prokaryotic gene function discovery are lacking. Here we present two prokaryotic CGP methods, based on phylogenetic profiles, to assist with this task.

**Results:**

Using gene occurrence patterns in sample genomes, we developed two CGP methods (statistical and inductive CGP) to assist with the discovery of bacterial gene functions. Statistical CGP exploits the differences in gene frequency against phenotypic groups, while inductive CGP applies supervised machine learning to identify gene occurrence pattern across genomes. Three rediscovery experiments were designed to evaluate the CGP frameworks. The first experiment attempted to rediscover peptidoglycan genes with 417 published genome sequences. Both CGP methods achieved best areas under receiver operating characteristic curve (AUC) of 0.911 in *Escherichia coli *K-12 (EC-K12) and 0.978 *Streptococcus agalactiae *2603 (SA-2603) genomes, with an average improvement in precision of >3.2-fold and a maximum of >27-fold using statistical CGP. A median AUC of >0.95 could still be achieved with as few as 10 genome examples in each group of genome examples in the rediscovery of the peptidoglycan metabolism genes. In the second experiment, a maximum of 109-fold improvement in precision was achieved in the rediscovery of anaerobic fermentation genes in EC-K12. The last experiment attempted to rediscover genes from 31 metabolic pathways in SA-2603, where 14 pathways achieved AUC >0.9 and 28 pathways achieved AUC >0.8 with the best inductive CGP algorithms.

**Conclusion:**

Our results demonstrate that the two CGP methods can assist with the study of functionally uncategorised genomic regions and discovery of bacterial gene-function relationships. Our rediscovery experiments also provide a set of standard tasks against which future methods may be compared.

## Background

Identifying gene functions is an important task in biology. The exponential growth of genome sequences has placed greater importance on the use of computational approaches for sequence analysis and annotation. With the development of high-throughput technology, methods of comparative genomics are increasingly used to assist with the identification of gene functions [1], as conventional methods of gene screening using transgenic organisms are resource intensive and time consuming. In practice, bench-side researchers frequently encounter extensive lists of genes that require further pruning and experimental validation. Accurate prioritisation of candidate genes, therefore, constitutes a key step in accelerating the discovery of gene functions.

*In silico *candidate gene prioritisation (CGP) ranks genes based upon the features associated with genes and the function of interest. A variety of *gene features *have been suggested for the prioritisation of causal genes in human diseases, including the co-occurrence of gene name and disease terminology in biomedical texts [2-5], sharing of terms in annotation or gene ontology databases [2,4,6-9], gene expression in different tissues [2,4,6], protein-protein interactions [4], similarity of gene or protein sequences [8,9], presence of genes within a phenotype or diseases database [10], phylogenetic relationships [11], or a combination of the above [2,4]. However, to construct a CGP system for prokaryotes, different forms of gene features are needed, as current CGP algorithms are skewed towards eukaryotic genomes and the systematic curation of annotation or genotype-phenotype databases are less complete than for eukaryotes. Hundreds of whole genome sequences of bacteria and thousands of partial genome sequences are available in public databases, yet prokaryotic genomes display a higher proportion of genes with unknown function than eukaryotes [12]. In contrast, several methods for computational protein function discovery have been studied, including chromosomal proximity method, domain fusion analysis, analysis of gene expression patterns, and phylogenetic profiles [13]. In particular, the phylogenetic profile method exploits knowledge of gene occurrences across a range of sequenced genomes and postulates that genes involved in the same metabolic pathway are frequently co-inherited. Phylogenetic profiles have been applied to unsupervised clustering of proteins to discover their functional linkages [14] and to discover conserved gene clusters in microbes (with probabilistic phylogenetic tree models) [15]. Supervised approaches of phylogenetic profiles have also been applied to infer protein networks (with canonical correlation analysis [16]) and predicting protein functional class in *Saccharomyces cerevisiae *(with tree-based kernels [17]), in the discovery of protein localisation in eukaryotes [18], in functional annotation of genes (by correlation enrichments [19]). These studies suggested that the concept of phylogenetic profiles provides a valuable tool for predicting gene-function linkage. It was thus hypothesised that such concept can also be exploited as *gene features *for prioritising genes contributing to a particular phenotypic trait of interest, thus providing a practical and generalisable tool to guide microbiologists in gene selection.

This paper examines the practical application of the phylogenetic profile method for gene prioritisation to investigate its generalisability and applicability on both simple and complex traits in prokaryotes.

Phylogenetic profiles form an indirect connection between gene and function in two conceptual steps. The first step establishes the gene-genome relationship, by examining the occurrence (presence or absence) of a candidate gene (or its homolog) in a given genome. The second step groups genomes according to their known phenotypes. We investigate two scenarios in which CGP can be useful in assisting with functional discovery of uncharacterised genes in prokaryotes. The method of *statistical *CGP is used when the occurrence profile can be directly inferred from the study phenotype, whereas *inductive *CGP is used when the profile is obscure but a small number of genes known to contribute to the study phenotype are available. Candidate genes are then prioritised by either statistical scoring functions or supervised machine learning algorithms.

In addition, at present there are no clear benchmarks to allow comparison between these different approaches to gene prioritisation, and the extent to which such algorithms are capable of identifying target genes in bacteria remains unexplored. This paper takes advantage of selected metabolic processes with a well-understood genetic basis to craft gold standard prioritisation tasks. The two CGP approaches are evaluated by rediscovering genes participating in well-characterised biochemical pathways – the metabolism of peptidoglycan, fermentation in anaerobes, and selected metabolic pathways curated in Kyoto Encyclopaedia of Genes and Genomes (KEGG) [20]. We ultimately aim to develop metrics that will provide an indication of the likelihood that highly prioritised genes are strong biological candidates, and the degree to all potential candidates have been identified for tasks such as the selection of biomarkers, the discovery of virulence genes, and the formulation of new hypotheses about uncharacterised genes.

## Methods

### Determination of genomic occurrences of candidate genes

To evaluate the performance of CGP methods, three case studies were selected for rediscovery experiments using well-known pathway genes as gold-standards. For each case study, the polypeptide sequences of *n *candidate genes were compared with all open reading frames (*orf*) of the *k *genome sequences from the National Centre for Biotechnology Information (NCBI, accessed April 2007) by Basic Local Alignment and Search Tool (BLASTP) . If a candidate gene reached the critical E-value of < 10^-5 ^in a given genome, a gene or gene homolog was defined as present in the genome. If a gene did not reach the critical E-value in a genome, the gene was recorded as absent from the genome. The binary states of gene occurrence were recorded in an *n *× *k *homolog matrix.

### Statistical CGP

From the *k *genomes, *k*_*p *_genomes known to display the phenotype of interest (*p*) were selected as positive genome examples, and *k*_*n *_genomes not displaying *p *were chosen as negative genome examples. For each of the *n *candidate genes, the number of co-presence (homologs present in positive genome examples) and co-absence (homologs absent in negative genome examples) were counted and presented into a 2 × 2 contingency table, from which a number of statistical scoring functions was calculated. The scoring functions included: a) sensitivity (*sens*, the proportion of genes present in the positive genome examples), b) specificity (*spec*, proportion of genes absent in the negative genome examples), c) positive and negative predictive values (*ppv/npv*, the proportion of positive/negative genomes were present/absent when the gene was present/absent), d) arithmetic (*amss*) and harmonic (*hmss*) mean of sensitivity and specificity, e) odds ratio (*OR*, the odds of a gene existed in the positive example versus the odds of a gene was absent in the negative examples), f) chi-square scoring function (*chisq*, the deviation of the observed frequency from the expected proportion), g) directional chi-square function (*bchisq*, the chisq function with genes that displayed inverse associations be reversed to the bottom of the rank), and h) F-measure (*F*, the harmonic mean of the sensitivity and precision). The mathematical definitions of these scoring functions are listed in the Additionl file 1.

### Inductive CGP

Inductive CGP ranks genes by finding genes with similar occurrence pattern across a number of bacterial genomes using supervised machine learning. A number of genes known to display a target phenotype or function *p *were selected as positive examples for the training set. Similarly, genes that did not contribute to *p *were selected as negative gene examples. The occurrences of genes in *k *genome examples were used as features for model training. Candidate genes were ranked by the score or posterior probability from the output of the machine learning classifiers. The machine learning classifiers included naïve Bayes (*NB*), logistic regression (*LR*; ridge = 10^-5^), *J48 *decision tree (*J48*, pruning confidence = 0.25), nearest neighbour classifier (*IBk*, with inverse distance weighing; *k *was determined by leave-one-out cross-validation), alternating decision tree (*ADTree*; boosting iteration = 10), support vector machines (*SVM*) with polynomial (*SVM/Poly*; linear kernel trained by sequential minimal optimisation algorithm, SMO) and radial basis function (*SVM/RBF*; trained by SMO; *γ *= 0.01) kernels. The Waikato Environment for Knowledge Analysis (WEKA) 3.5.6 was used for classifier training [21]. For the purpose of benchmarking, the generalisation performance of inductive CGP was evaluated by stratified 10-fold cross-validation: for the *n *genes used as candidate genes for prioritisation, all *n*_+ _genes from the validation set and the rest of *n*_- _genes not in the validation set were each randomly divided into 10 subsets. One-tenth of the the genes from each group ($\frac{1}{10}$ of *n*_+ _and *n*_- _genes) were sequentially selected as test set, whereas the rest of the genes were selected as training set to train inductive models. The performance of each inductive CGP algorithm was obtained by averaging areas under receiver operating characteristic curves (AUC) over the 10 runs.

### Evaluation of CGP performance

The performance of different CGP methods was evaluated by rediscovery experiments. The relative position of the ranked candidate gene was measured by percentiles from the top of the rank (*pct*). The AUCs were estimated non-parametrically by trapezoidal rule. We adopted probability enrichment (the relative enrichment ratio) described by Turner et al [7] to compare the performance of different statistical CGP scoring functions [see Additional file 1]. The average and maximum probability enrichments, defined as *n *folds-improvement in precision above a certain score threshold *τ*, were calculated by partial precision (*pppv*), such that:

$$\begin{array}{lll} \text{Partial prec}.\text{ at }\tau & = & pppv(\tau )\\ & = & \frac{\text{Num}.\text{ correct genes}>\tau }{\text{Num}.\text{ genes}>\tau } \end{array}$$

and the average ($\bar{\eta }$) and the maximum (*η*_*max*_) probability enrichments were defined as:

$$\begin{array}{c} \eta (t)=\frac{pppv_{n}(t)}{ppv}\\ \bar{\eta }=\int_{0}^{1}\eta (t)dt\\ \eta_{max}=\eta (t^{*}) \end{array}$$

where *t *was the rank fraction at threshold *τ*, *ppv *was the overall precision, *pppv*_*n*_(*t*) was the partial precision at rank fraction *t*, and *η*(*t*) was at its maximum at t*. Both AUC and  measure the overall performance of a CGP task. The rank fraction t* indicates the point above which correct genes are likely to be found *η*_*max*_-times more likely than compared to a random gene list. Evaluation with *η*_*max *_is useful to identify cases where a small proportion of genes is ranked highly but the overall performance is poor.

### Effect of number of genome examples on CGP performance

Two simulation experiments were performed to investigate the effect of the number of genome examples on statistical CGP performance (Case study 1, see below). For the first simulation, the *amss *scoring function was repeatedly applied on randomly selected subsets of 417 positive and negative genome examples, using genes from set M (Case study 1). The number of positive genome examples (*N*_*p*_) and negative genome examples (*N*_*n*_) were gradually increased in each subset. For each combination of *N*_*p *_and *N*_*n*_, 25 runs were performed and the median AUC was obtained. A second simulation was performed to determine the variability of performance. Here the proportion of positive and negative genome examples was kept the same (400:17) and the median and the range of AUC were then obtained over 1000 runs for each *N*_*p *_and *N*_*n*_.

A similar simulation was also performed to determine the effect of genome example sizes on inductive CGP performance. Twenty five subsets of *N *genomes (from 417 genomes) were randomly selected as features with *N *increased from 1 to 417. For each *N*, stratified 10-fold cross-validations were performed with *SVM/Poly *using all genes from SA-2603 genome as candidates. Median AUCs from 25 random subset of *N *genomes were obtained.

### The case studies

#### Case study 1: Identification of genes involved in bacterial cell wall synthesis

Well-characterised genes responsible for peptidoglycan biosynthesis and metabolism in bacteria were used for testing and were grouped into three nested validation sets [see Additional file 2]. The *C *(core) validation set consisted of genes responsible for the synthesis of *N*-acetylmuramate-pentapeptide from UDP-*N*-acetylglucosamine (*mur*A to *mur*G, and *mra*Y). The *B *(biosynthesis) validation set, extended the *C *set with genes involved in precursor pathways including *N*-acetyl-D-glucosamine, *meso*-diaminopelamate and D-alanyl-D-alanine, as well as genes responsible for undecaprenyl phosphate biosynthesis and recycling. The *M *(metabolism) validation set further extended the *B *set by including genes responsible for the modification, recycling, and cross-linking of the peptidoglycan such as penicillin-binding proteins and *N*-acetylmuramoyl-L-alanine amidases [see Additional file 3]. Genome examples were selected from the NCBI bacterial genomes catalogue file [22] and manually verified by one of the authors (RL). Genes in the validation sets were identified using KEGG [20] and EcoCyc [23]. Genomes of one Gram positive bacterium (*S. agalactiae *2603 V/R, SA-2603, 2124 genes, GenBank ID: AE009948) and one Gram negative bacterium (*E. coli *K-12, EC-K12, 4134 genes, GenBank ID: U00096) were selected for prioritisation.

For statistical CGP, 400 genomes of bacteria known to produce peptidoglycan were selected as positive examples. Genomes of 17 bacterial species lacking cell wall, including *Mycoplasma *spp., *Ureaplasma *spp., *Anaplasma *spp., and *Phytoplasma *spp., were selected as negative examples [see Additional file 4]. For inductive CGP, the occurrence of the candidate genes in the same 417 genomes was used as features for machine learning training. Candidate genes were labelled according to whether they belong to *C*, *B*, and *M *validation sets. To compare the effectiveness of statistical CGP, the relative positions of the peptidoglycan genes were compared with an unrelated metabolic pathway (glycolysis genes) acting as the control validation set [see Additional file 5].

#### Case study 2: Anaerobic mixed acid fermentation genes

Enzymes responsible for anaerobic respiration and fermentation were identified from pathway databases [20,23] and literature searches [24,25]. Statistical and inductive CGP methods were used to derive the occurrence matrix and to rank candidate genes for anaerobic mixed-acid fermentation in EC-K12. All genes in EC-K12 were used as candidates for prioritisation. For statistical CGP, 200 bacterial genomes of known obligatory and facultative anaerobes capable of performing anaerobic metabolism were selected as positive genome examples, and 142 genomes of obligatory aerobes that do not perform anaerobic respiration were applied as negative examples [see Additional file 6]. Methods for genome example selection were identical to Case study 1. For inductive CGP, the occurrence patterns of 4134 candidate genes in 342 genomes were obtained by the methods described above.

#### Case study 3: KEGG Pathways

To evaluate the generalisability of inductive CGP, a large-scale rediscovery experiment based on the curated KEGG metabolic pathways was performed [20]. Thirty-one metabolic pathways with at least 10 genes involved in each pathway were selected for evaluation from the 81 known pathways available for the SA-2603 genome in KEGG. All seven inductive CGP algorithms were tested, and the generalisation performance of the algorithms evaluated by stratified 10-fold cross-validation.

## Results

### Case study 1: Peptidoglycan-related genes

**Figure 1:**
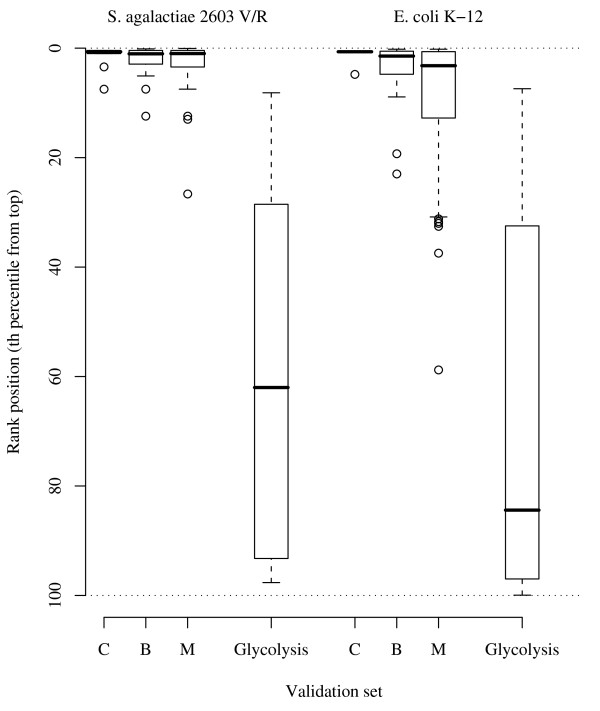
**The performance of statistical CGP in rediscovering peptidoglycan-related genes (amss scoring function)**. The box-and-whisker plot shows the result of statistical CGP (*amss *scoring function) in rediscovering peptidoglycan-related genes in the two study genomes (*Streptococcus agalactiae *2603 and *Escherichia coli *K-12). The horizontal bars indicate medians of the prioritised ranks and the boxes indicate the upper and lower quartiles. Groups *C*, *B*, and *M *indicate 3 sets of validation genes used. Genes from the glycolytic pathways were used as controls for comparison.

The best scoring functions for rediscovering metabolic genes (*M *set) were the *amss*, *hmss*, and *npv *(AUC >0.970) using the whole genome of SA-2603 (2124 genes) as candidate genes. Of the 25 known peptidoglycan-related genes, all except one gene were identified within the top 13% (median: top 1.0 *pct*) in SA-2603 (Table 1 and Figure 1). The top-scored genes in the SA-2603 genome are listed in [see Additional file 7]. Encouraging results were also achieved in prioritising the EC-K12 genes in all three validation sets (Table 2); for example, for the *M *set genes in EC-K12 (51 known genes out of 4134 genes in the bacterial genome), an AUC of 0.911 was achieved by *amss*, and the median of the rediscovered genes was at the top 3.2 *pct *of the rank. In contrast, poor performances were yielded when matching the control validation sets (glycolysis) against the same *amss*-prioritised ranks (SA-2603: 0.398; EC-K12: 0.341; Figure 1).

**Table 1:** CGP performance on peptidoglycan-related genes (*Streptococcus agalactiae *2603 V/R, 2124 genes).

	Validation sets					
	
Methods	C (9 genes)		B (18 genes)		M (25 genes)	
			
	AUC	(/*η*_*max*_)	AUC	(/*η*_*max*_)	AUC	(/*η*_*max*_)
Statistical CGP (scoring functions)						
*sens*	0.858	(2.0/5.4)	0.853	(1.9/4.3)	0.830	(1.8/3.8)
*spec*	0.396	(0.5/1.5)	0.427	(0.7/2.6)	0.506	(1.1/5.2)
*ppv*	0.420	(0.6/1.57)	0.504	(1.3/29.5)	0.590	(2.1/85.0)
*npv*	0.966	(3.6/30.1)	0.964	(3.5/21.7)	0.978	(3.2/17.3)
*amss*	0.985	(4.6/88.5)	0.980	(4.4/59)	0.970	(4.4/85.0)
*hmss*	0.986	(4.8/88.5)	0.980	(4.5/64)	0.969	(4.5/85.0)
*OR*	0.415	(0.5/1.57)	0.509	(1.3/29.5)	0.592	(2.1/85.0)
*chisq*	0.978	(4.2/59.0)	0.975	(3.9/34.7)	0.959	(3.7/28.3)
*bchisq*	0.978	(4.2/59.0)	0.975	(3.9/34.7)	0.960	(3.7/28.3)
*F*	0.932	(3.3/32.9)	0.915	(3.1/23.1)	0.881	(2.8/18.5)
						
Inductive CGP (machine learning algorithms)						
*NB*	0.901		0.879		0.843	
*LR*	0.980		0.905		0.887	
*ADTree*	0.996		0.944		0.975	
*IBk*	0.948		0.950		0.974	
*J48*	0.885		0.832		0.752	
*SMO/Poly*	0.999		0.948		0.879	
*SMO/RBF*	0.998		0.991		0.909	

This table lists the performance of statistical and inductive CGPs in prioritising peptidoglycan-related genes in *Streptococcus agalactiae *2603 V/R. Abbreviations: *sens*: sensitivity; *spec*: specificity; *ppv*: positive predictive value; *npv*: negative predictive value; *amss*: arithmetic mean of sensitivity and specificity; *hmss*: harmonic mean of sensitivity and specificity; *OR*: odds ratio; *chisq*: chi-square; *bchisq*: signed chi-square; *F*: F-measure; *NB*: naïve Bayes classifier; *LR*: logistic regression; *ADTree*: alternating decision tree; *IBk*: k-nearest neighbour classifier; *J48*: *J48 *decision tree; *SMO*: support vector machine trained by sequential minimal optimisation algorithm; *Poly*: polynomial kernel; *RBF*: radial basis function kernel.

**Table 2:** CGP performance on peptidoglycan-related genes (*Escherichia coli *K-12, 4131 genes).

	Validation sets					
	
Methods	C (8 genes)		B (28 genes)		M (51 genes)	
			
	AUC	(/*η*_*max*_)	AUC	(/*η*_*max*_)	AUC	(/*η*_*max*_)
Statistical CGP (scoring functions)						
*sens*	0.913	(2.5/10.6)	0.891	(2.3/6.0)	0.818	(1.9/4.2)
*spec*	0.321	(0.4/1.4)	0.310	(0.4/1.2)	0.418	(0.8/2.0)
*ppv*	0.405	(0.8/5.2)	0.423	(1.2/18.4)	0.553	(1.7/28.6)
*npv*	0.974	(3.9/42.0)	0.956	(3.5/20.9)	0.891	(2.8/13.2)
*amss*	0.989	(4.8/110.)	0.966	(4.1/53.7)	0.911	(3.5/44.7)
*hmss*	0.989	(4.9/113.)	0.969	(4.2/55.3)	0.909	(3.5/45.6)
*OR*	0.403	(0.8/5.2)	0.424	(1.2/18.4)	0.552	(1.7/28.6)
*chisq*	0.984	(4.7/73.8)	0.963	(3.9/35.9)	0.902	(3.2/27.0)
*bchisq*	0.984	(4.7/73.8)	0.963	(3.9/35.9)	0.903	(3.2/27.0)
*F*	0.965	(4.0/45.8)	0.921	(3.2/22.5)	0.838	(2.5/15.1)
						
Inductive CGP (machine learning algorithms)						
*NB*	0.930		0.889		0.820	
*LR*	0.882		0.935		0.828	
*ADTree*	0.976		0.981		0.925	
*IBk*	0.998		0.929		0.946	
*J48*	0.935		0.828		0.752	
*SMO/Poly*	0.997		0.876		0.933	
*SMO/RBF*	0.963		0.932		0.964	

This table lists the performance of statistical and inductive CGP in prioritising peptidoglycan-related genes in *Escherichia coli *K-12. Abbreviations: *sens*: sensitivity; *spec*: specificity; *ppv*: positive predictive value; *npv*: negative predictive value; *amss*: arithmetic mean of sensitivity and specificity; *hmss*: harmonic mean of sensitivity and specificity; *OR*: odds ratio; *chisq*: chi-square; *bchisq*: signed chi-square; *F*: F-measure; *NB*: naïve Bayes classifier; *LR*: logistic regression; *ADTree*: alternating decision tree; *IBk*: k-nearest neighbour classifier; *J48*: *J48 *decision tree; *SMO*: support vector machine trained by sequential minimal optimisation algorithm; *Poly*: polynomial kernel; *RBF*: radial basis function kernel.

The performance of statistical CGP was also measured by folds-increase in precision (probability enrichments) compared to the non-prioritised rank. With the *chisq *scoring function, the ranked gene list achieved an average enrichment of 3.65 folds (maximum 28.3 folds) for SA-2603 and 3.16 folds for EC-K12 (maximum 27 folds). The probability enrichments of other validation sets are listed in Tables 1 and 2. High AUC values were obtained from the stratified cross-validations of inductive CGP experiments. In particular, *SVM *achieved near-perfect AUCs in both SA-2603 *C *and *B *validation sets, whereas *ADTree *had the best AUC of 0.975 in *M *set genes (the trained *ADTree *model is shown in Additional file 8). Similarly, the best AUC was achieved by *SVM/RBF *in the EC-K12 *M *validation set (0.964). The best AUCs of 0.998 and 0.981 were also achieved in the rediscovery of *C *and *B *set genes (by *IBk *and *ADTree *respectively).

**Figure 2:**
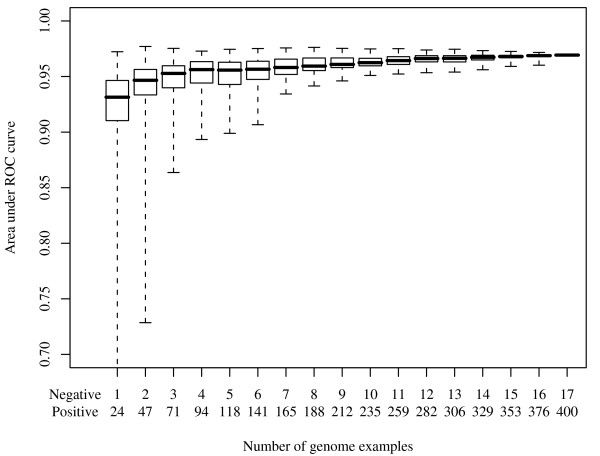
**AUC versus number of genome examples in statistical CGP on Streptococcus agalactiae 2603 peptidoglycan-related genes (amss scoring function)**. This figure demonstrates how the number of genome examples may influence statistical CGP performance (*amss *scoring function). The proportion of positive:negative genome examples were fixed (400:17).

**Figure 3:**
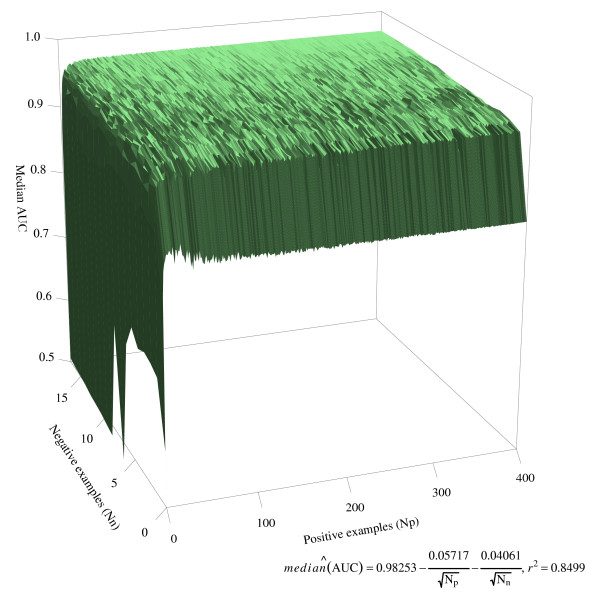
**Three-dimensional surface plot demonstrating the effect of number of genome examples on the statistical CGP performance**. The median AUCs (*z*-axis) over 25 simulation runs are shown for each *N*_*p *_(*x*-axis) and *N*_*n *_(*y*-axis) combination. Statistical CGP (*amss *scoring function) was performed to discover peptidoglycan-related genes by prioritising all genes in the *Streptococcus agalactiae *2603 genome.

Simulations were performed to investigate the effect of number of genome examples on statistical CGP performance. The range of AUCs was found to be considerably broader with fewer genome examples (Figure 2). However, a median AUC (>0.95) could still be achieved with as few as 10 genome examples in each group in the rediscovery of the *M *set genes, compared with a maximum of 0.97 using all 417 bacterial genomes (Figure 3), indicating that the method has considerable power using even a very small genome sample.

**Figure 4:**
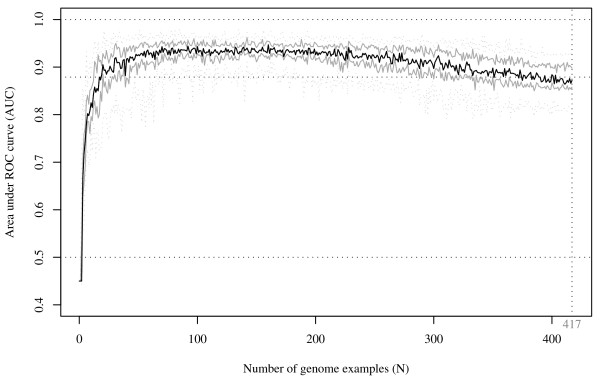
**Line plot illustrating the effect of number of genome examples on the inductive CGP performance**. This figure shows the effect of AUC versus number of genome examples in inductive CGP (*SVM/Poly *algorithm) on *Streptococcus agalactiae *2603 genes related to peptidoglycan metabolism (M validation set). Twenty-five simulation runs of stratified 10-fold cross-validation were performed. The black line indicates the median AUC, the grey solid lines indicate the upper and lower quartiles, and the dotted grey lines indicate the maximum and minimum AUC of the simulation runs.

The same simulation performed on inductive CGP (with *SVM/Poly*) also achieved an median AUC >0.90 with only 20 genome examples (Figure 4). It was noted, however, that the AUCs peaked at 70 to 160 genome examples (with corresponding AUCs between 0.93–0.95) and the performance gradually declined as more genome examples were added to the profile. Considerable variation of AUC was also noted when the full 417 genomes were included in the profile panel (median AUC: 0.872; interquartile range: 0.858–0.898; range: 0.824–0.925).

### Case study 2: Anaerobic mixed-acid fermentation genes

Statistical CGP on the anaerobic mixed-acid fermentation rediscovery task for EC-K12 performed poorly (AUC: 0.46–0.77). However, the maximum probability enrichment was high (up to 108-folds, Table 3). Bacterial genes specific to anaerobic metabolism were identified with high ranking scores (the *pfl *complex: above 0.27 *pct*; *adh*E: 2.1 *pct*; *ack*A: 2.1 *pct*; *pta*: 12 *pct*; see Additional files 9 and 10) by *amss*. In contrast, genes shared with aerobic respiration, such as the fumerase genes (*fumABC*) and the phosphoenolpyruvate carboxylase gene (*ppc*), were ranked much lower (61–96 *pct *and 57 *pct *respectively). For genes encoding the fumarate reductase complex, there were mixed results: the membrane anchor subunits (*frd*CD) were ranked highly (10.1 and 7.8 *pct *respectively) and the catalytic subunits were placed at the bottom of the rank (*frd*AB, 93 and 99 *pct*). Better overall performance of inductive CGP was achieved compared with statistical CGP (AUC: 0.70–0.86). The best AUCs with inductive prioritisation were produced by *IBk *and *SVM/Poly *algorithms respectively (0.86 and 0.85).

**Table 3:** CGP performance on anaerobic mixed-acid fermentation genes (Escherichia coli K-12, 4131 genes). Prioritisation (AUC) of anaerobic mixed-acid fermentation genes in *Escherichia coli *K-12

Statistical CGP			Inductive CGP	
	
Scoring function	AUC	(/*η*_*max*_)	Algorithm	AUC
*sens*	0.634	(1.2/1.8)	*NB*	0.695
*spec*	0.464	(0.8/1.5)	*LR*	0.796
*ppv*	0.519	(1.1/2.0)	*ADTree*	0.780
*npv*	0.594	(1.8/11.0)	*IBk*	0.860
*amss*	0.578	(2.4/96.6)	*J48*	0.663
*hmss*	0.628	(2.4/95.1)	*SMO/Poly*	0.848
*OR*	0.537	(1.2/2.3)	*SMO/RBF*	0.782
*chisq*	0.767	(3.2/109)		
*bchisq*	0.585	(2.5/109)		
*F*	0.698	(2.5/69.9)		

Thirty-eight known genes were labelled as known (out of 4131 genes of the EC-K12 genome). The AUC in inductive CGP were calculated using stratified 10-fold cross-validation. Abbreviations: *sens*: sensitivity; *spec*: specificity; *ppv*: positive predictive value; *npv*: negative predictive value; *amss*: arithmetic mean of sensitivity and specificity; *hmss*: harmonic mean of sensitivity and specificity; *OR*: odds ratio; *chisq*: chi-square; *bchisq*: signed chi-square; *F*: F-measure; *NB*: naïve Bayes classifier; *LR*: logistic regression; *ADTree*: alternating decision tree; *IBk*: k-nearest neighbour classifier; *J48*: *J48 *decision tree; *SMO*: support vector machine trained by sequential minimal optimisation algorithm; *Poly*: polynomial kernel; *RBF*: radial basis function kernel.

### Case study 3: Inductive prioritisation of KEGG pathway genes

**Figure 5:**
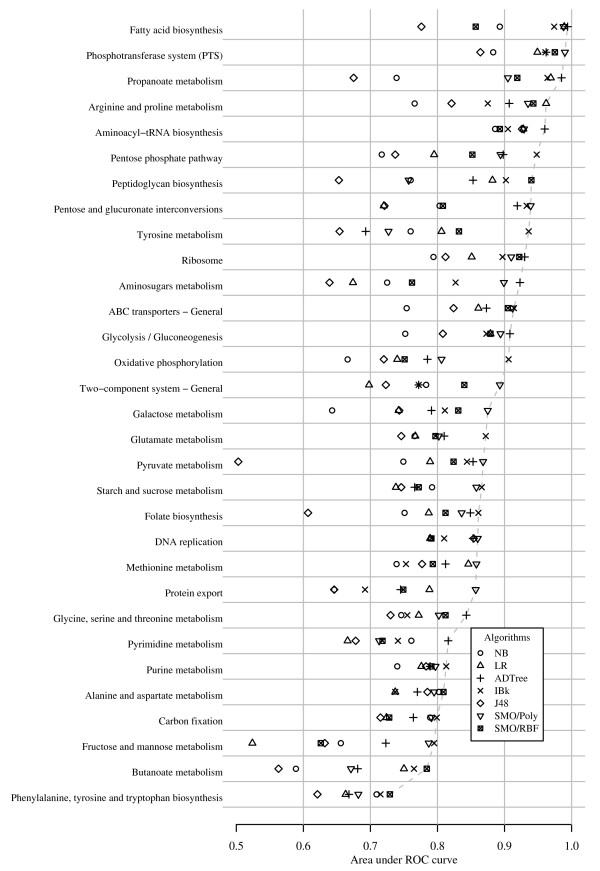
**The performance of inductive CGP in prioritising 31 KEGG metabolic pathways**. The AUCs attempted by stratified 10-fold cross-validations were obtained by the rediscovery experiment in Case study 3. Genes of 31 metabolic pathways of *S. agalactiae *2603 genome were obtained from KEGG and rediscovered by 7 machine learning algorithms.

Inductive CGP was conducted on 31 KEGG pathways of SA-2603 using 7 algorithms. The best supervised machine learning algorithms identified 14 pathways (45%) with AUCs >0.90 and 28 pathways (87%) with AUCs >0.80 (Figure 5 and see Additional file 11). The best performing algorithm was *IBk *which had the highest AUC in 10 pathways. *ADTree *and *SVM/Poly *also performed well, with each producing the best AUC in 8 pathways. *SVM/RBF *achieved best AUC in 4 pathways. *NB *and *J48 *did not produce a best AUC in any of the 31 pathways studied.

## Discussion

### Successful prioritisation of bacterial genes by occurrence-based CGP methods

In this paper, we applied two approaches (statistical and inductive CGP) to prioritise candidate genes for functional discovery, based on the occurrence patterns of candidate genes in a selected set of bacterial genomes (phylogenetic profiles). Our findings demonstrate that both CGP methods can rediscover genes with high accuracy in two selected genomes of *E. coli *K-12 and *S. agalactiae *2603 (Figure 3).

Interestingly, these methods seem relatively insensitive to the number of genome examples. In the peptidoglycan example with statistical CGP (case study 1), we were able to identify peptidoglycan genes with high accuracy, despite only a limited number of sequenced genomes among negative examples. For inductive CGP, increasing profile dimension beyond 200 genome examples apparently resulted in a decrease in subsequent median AUCs, implying that a proportion of genome examples was less informative and might not have contributed to the identification of genes of interest. This finding coincides with the observation by Johti *et al.*, where increasing the phylogenetic profile dimension with redundant genomes did not necessarily improve the accuracy in eukaryotic gene function prediction [26].

In statistical CGP, we found that the scoring functions measuring gene occurrence in both positive and negative genome example groups (*amss*, *hmss*, *chisq*, and *bchisq*) consistently outperform the scoring functions that measure only positive (*sens *and *ppv*), negative (*spec *and *npv*), or partial (*F*-measure) frequencies of the groups. This finding highlights the importance of including both positive and negative examples in comparative genomic studies. In inductive CGP, the rediscovery experiments favoured *ADTree*, *IBk*, and *SVM *s when compared with other algorithms. In case study 1, the performance of the best inductive and statistical CGP methods are comparable, suggesting that both approaches are capable of producing robust results.

### Statistical CGP rediscovers genes specific to the function of interest

Our results demonstrated that statistical CGP can discover genes specific to a particular function or pathway. For example, by comparing the cell-walled bacteria with cell wall-less *Mycoplasma *spp. (case study 1), it was expected that the peptidoglycan genes, which are specific for the phenotypic trait, were among the genes lost in this evolutionary lineage [27] (See Additional file 12). Such genes were ranked very highly and yielded favourable aggregated performance (with AUC >0.95). In contrast, results from our anaerobic fermentation experiment (case study 2) suggested that genes specific to anaerobic respiration were placed very highly on the rank (*η*_*max *_> 95-fold), whereas genes *sharing *with the obligatory aerobic bacteria (negative examples) were ranked much lower. As these shared genes are present in both phenotypic groups, finding these "shared" genes by applying only statistical CGP is a challenging task. Alternative methods are needed to aid in the discovery of such non-specific genes.

### Evolutionary pressure may contribute to specific gene occurrence patterns

Both occurrence-based CGP methods performed well, suggesting the genes encoding for a complex phenotype are frequently co-present and co-absent across the genomes, forming specific occurrence patterns, thus allowing the functional predictions. This co-occurrence phenomenon may reflect the process of natural selection and the adaptation of microorgranisms into different evolutionary niches.

For bacteria undergoing positive selection, the acquisition of a particular gene group may result in phenotypes conferring survival advantage for the microorganism to adapt to a new environment. It has been known that genes contributing to symbiosis or pathogenesis are frequently organised into genomic islands, in which gene mobility is facilitated by horizontal gene transfer, conferring the ability to form a new relationship with the host [28]. The good AUC achieved by inductive CGP in KEGG pathways (case study 3) suggests specific functional co-occurrence patterns of genes do exist, regardless of the physical proximity of the genes or the presence of a mobile genetic structure.

Similarly, negative selection can also contribute to the co-absence of functional gene units across multiple genomes. For a complex phenotype encoded by multiple genes, the deletion of a critical gene could result in the non-expression of phenotype, leading to the subsequent loss of other non-functional genes over time. Thus, the differential co-occurrence patterns in genes can be exploited for comparative genomics studies, as demonstrated by our methods, in assisting our understanding of gene functions.

### Factors affecting CGP performance

#### Sampling biases

Prioritising candidate genes with reliance on gene features from literature, ontology, or annotation as background knowledge may introduce literature or annotation biases [2,4,9]. While we minimised such biases, several sources of *sampling bias *could have limited the performance of our gene prioritisation methods. With statistical CGP, accuracy can be affected by *ad hoc *selection of examples, especially by the choice of positive and negative genome examples representing the variations in the study phenotype. With inductive CGP, performance may be impeded by incorporating inconsistent (or wrong) genes in the training set. Increasing the heterogeneity of the training genes may adversely influence prioritisation performance. An example can be found in a slight decrease of performance (in median AUC) from *C *to *M *validation sets in the peptidoglycan experiments in EC-K12 candidates, as shown in Figure 1.

#### Using KEGG as a validation data source

There were considerable variations in inductive CGP performances across different KEGG categories (Case study 3). By manually inspecting the worst-performing functional category (phenylalanine, tyrosine, and tryptophan biosynthesis), we found the phenylalanine and tyrosine tRNA synthases genes were also included in the validation set. The tRNA synthases have roles downstream of the biosynthesis pathways and thus are not involved in the anabolism of these essential amino acids. Removal of the unrelated genes improved overall performance (with best AUC of 0.852 achieved by *SVM/Poly*, see Additional file 13). This contrasts with the best-performing pathways (for example, fatty acid biosynthesis and peptidoglycan synthesis pathways) where only function-specific genes were included in the validation set. Since KEGG is a commonly-used resource for benchmarking computational methods of functional discovery [15,16,26], our finding suggests that careful selection must be practised in constructing validation sets, as mixture of distinct functional groups could lead to inconsistent results. This specific sampling bias needs to be considered when explaining variations in the predicting of gene functions by *in silico *methods.

#### The inclusion of paralogs in the occurrence matrix

Reciprocal best BLAST matches are frequently used in the search for orthologous genes. In our experiments, we applied non-reciprocal BLAST E-value < 10^-5 ^as the criterion for determining the sequence similarity between genes. While our results supported its use in functional discovery at the gene level, the use of such criterion may include many paralogs and may affect prioritisation performance of large gene families with diverse functions. Detecting and excluding paralogs may be required to refine the gene ranking and warrant further studies.

## Conclusion

We developed a statistical and an inductive computational gene prioritisation methods, based on the concept of gene occurrence across a range of genomes, to improve the search efficiency in the functional discovery of bacterial genes. We designed a range of rediscovery experiments for benchmarking different CGP approaches. Promising results were yielded from the testing on the rediscovery of peptidoglycan-related genes, mixed-acid fermentation genes, and a diverse range of bacterial metabolic pathways. These CGP methods could be generalised to other functional discovery tasks when a pair of positive and negative datasets are available (statistical CGP) or when a subset of genes with known functions can be used for training machine learning models (inductive CGP). With more genome sequences become available, we anticipate the demand of such methods will grow as many different scenarios can be formulated and analysed. In summary, occurrence-based gene prioritisation method offers a simple yet effective framework for ranking candidate genes for functional discovery in prokaryotes. In addition, our experimental framework should provide a standardised benchmark for evaluating future CGP methods and algorithms when prioritising bacterial candidate genes.

## Competing interests

The authors declare that they have no competing interests.

## Authors' contributions

FL contributed to the conception of the CGP frameworks and performed the rediscovery experiments. EC and RL contributed to the experimental design. All authors (FL, EC, RL, and VS) contributed to the critical analysis, interpretation, and discussion. All authors contributed to the preparation of manuscript. All authors read and approved the final version of the manuscript. We thank the anonymous reviewers for their valuable comments and criticisms.

## Supplementary Material

Additional file 1 **This file lists the mathematical definitions of the statistical scoring functions evaluated in Case studies 1 and 2.** Click here for file

Additional file 2 **The *C *validation set (shaded area) includes genes responsible for synthesis of the peptidoglycan backbone (shaded area).** The *B *validation set includes various accessory pathways (UDP-NAG synthesis, D-Glu and D-Ala synthesis, *meso*-DAP synthesis, and und-PP synthesis and recycling). The *M *validation set further includes genes responsible for transpeptidation, transglycosylation, and other genes responsible for peptidoglycan metabolisms. Abbreviations: UDP: uridine diphosphate; NAG: *N*-acetylglucosamine; NAG-1P: *N*-acetylglucosamine-1-phosphate; NAM: *N*-acetylmuramate; NAG-EP: *N*-acetylglucosamine-enopyruvate; Ala: alanine; Glu: glutamate; (D-Ala)_2_: D-alanyl-D-alanine; m-DAP: *meso*-diaminopelamate; Und-PP: undecaprenyl diphosphate; Und-P: undecaprenyl phosphate; F6P: fructose-6-phosphate; D-Glc: D-glucosamine; D-Glc-6P: D-glucosamine-6-phosphate; D-Glc-1P: D-glucosamine-1-phosphate; L-Asp: L-aspartate; L-Asp-4P: L-aspartate-4-phosphate; ASA: aspartate semialdehyde; DHDP: L-2,3-dihydrodipicolinate; THDP: tetrahydrodipicolinate; NS-AKP: *N*-succinyl-2-amino-6-ketopimelate; NS-DAP: *N*-succinyl-L,L-2,6-diaminopimelate; L,L-DAP: L,L-diaminopimelate. Click here for file

Additional file 3 **The genes and the validation sets of peptidoglycan-related genes used in Case study 1.** Click here for file

Additional file 4 **This file lists the 400 positive and 17 negative genome examples used in statistical CGP of peptidoglycan-related genes.** Click here for file

Additional file 5 **This file lists the positions of glycolysis genes in the ranks produced by statistical CGP of peptidoglycan genes.** Click here for file

Additional file 6 **This file lists the 200 positive and 142 negative genome examples used in statistical CGP of anaerobic mixed-acid fermentation genes.** Click here for file

Additional file 7 **The rank positions, rank fractions (in *pct*), cluster of orthologous groups (COG), and the positions of candidate genes in the reference genome (SA-2603) ranked by *amss *scoring function.** Click here for file

Additional file 8 **This figure shows the alternating decision tree (*ADTree*) model induced by M-validation set of SA-2603 genome.** This model predicts whether a gene is related to peptidoglycan metabolism by summing the scores of all preceding nodes from root (Start). A higher score would rank the candidate gene higher. The model shown in this figure achieved an AUC of 0.975 as estimated by using stratified 10-fold cross-validation. Abbreviations of genome names: Nit. europ.: *Nitrosomonas europaea *(GenBank accession: AL954747); Wig. brevipalpis.: *Wigglesworthia brevipalpis *(AB063523, BA000021); Oen. Oeni PSU-1: *Oenococcus oeni *PSU-1 (CP000411); Clos. tetan. E88: *Clostridium tetani *E88 (AE015927, AF528097); Myc. mycoides.: *Mycoplasma mycoides *(BX293980); Ehr. ruminantium str.: *Ehrlichia ruminantium str*. Welgevonden (CR925678); Buc. aphidicol. Cc Cinara cedri.: *Buchnera aphidicola *Cc Cinara cedri (CP000263); Hah. chejuensis: *Hahella chejuensis *KCTC 2396 (CP000155); Ric. felis URRWXCal2: *Rickettsia felis *URRWXCal2 (CP000053–CP000055); Por. gingivalis. W83: *Porphyromonas gingivalis *W83 (AE015924) Click here for file

Additional file 9 **The rank fraction (in *pct*) of genes prioritised by *amss *scoring function in the EC-K12 genome.** Click here for file

Additional file 10 **The rank positions, rank fractions (in *pct*), cluster of orthologous groups (COG), and the positions of candidate genes in the reference genome (*E. coli *K-12) prioritised by *amss *scoring function.** Click here for file

Additional file 11 **This is the tabular representation of results in Figure**5**, showing the AUCs of 10-fold cross-validations in rediscovering genes in the 31 KEGG pathways evaluated in Case study 3.** Click here for file

Additional file 12 **In Case study 1, evaluation experiments were performed on candidate genes selected from one *S. agalactiae *and one *E. coli *genomes.** These bacterial genomes belong to divisions of Firmicutes and Gamma-proteobacteria, both consisting of large number of closely-related sequences in positive examples, and it could have favourably biased the performance due to over-representation. This file describes an additional CGP experiment by selecting a less-well represented genome from the NCBI database, *Prochlorococcus marinus *MIT9313, to investigate this effect. Click here for file

Additional file 13 **There were considerable variations in inductive CGP performance in Case study 3, and some variations is attributable to statistical uncertainties or algorithmic differences.** The influence of pathway functions on CGP performance was, however, unclear. Nevertheless, it was observed that there may be limitations in using KEGG pathways as a validation source, where potential sampling biases could have explained a significant proportion of such variations. In this file, an additional experiment was performed to illustrate this effect. Click here for file
