# Supplementary material for: In silico prioritisation of candidate genes for prokaryotic gene function discovery: an application of phylogenetic profiles
Source: BMC Bioinformatics. 2009 Mar 17;10:86. doi: 10.1186/1471-2105-10-86 (PMC2669486; doi:10.1186/1471-2105-10-86)
Supplement: Additional file 1 — This file lists the mathematical definitions of the statistical scoring functions evaluated in Case studies 1 and 2. [file 1471-2105-10-86-S1.pdf]

Additional file 1 — Scoring functions applied in statistical CGP

- *Gene* g_i: a gene
- *Strain* s_j: a bacterial isolate
- *Genome* $\mathbf{G}_j$: The corresponding genome of s_j, which consists of the entire set of genes such that

$$s_j \overset{\text{has}}{\rightarrow} \mathbf{G}_j = \{g_{j_1}, g_{j_2}, \ldots, g_{j_m}\}$$

- *Gene product* y_k: the product of a gene (i.e.. protein or RNA). For every gene product y_k, there exists at least one encoding gene g_i

$$g_i \Rightarrow y_k$$

- *Gene equivalence*: Two genes are considered equivalent if genes g_i and g_j both encode for the same gene product y_k, i.e.

$$g_i \equiv g_j \text{ if } g_i \Rightarrow y_k \text{ and } g_j \Rightarrow y_k$$

- *Set of gene products*: gene products of strain s_j

$$\mathbf{G}_j \overset{\text{encodes for}}{\rightarrow} \mathbf{Y}_j = \{y_{j1}, y_{j2}, \ldots, y_{jn},\}$$

- *Phenotype or function* p: the phenotypic expression of a bacterial strain
- *Phenotypic examples* $\mathbf{E}_p$: For each phenotype p, a list of phenotypic examples can be gathered. Each e_j correspond to a bacterial strain s_j.

$$\mathbf{E}_p = \{e_{p_1}, e_{p_2}, \ldots, e_{p_n}\}$$

  where $e_{p_k} \in \{s_{p_1}, s_{p_2}, \ldots, s_{p_n}\}$, are selected from bacterial strains display phenotype p.

Scoring functions

Sensitivity (*sens*) and specificity (*spec*)

Sensitivity is the proportion of candidate genes g present in genome $\mathbf{G}$ displaying phenotype p, whereas specificity is the proportion of genes g absent in genomes $\mathbf{G}$ that also do not display p. These measures are equivalent to the normalised rate of *co-presence* and *co-absence* of genes in the positive and negative genome examples respectively:

$$\begin{aligned} sens(g) &= P(g|\mathbf{G} \in \mathbf{E}_p^+) = \frac{TP}{TP + FN} \\ spec(g) &= P(\neg g|\mathbf{G} \in \mathbf{E}_p^-) = \frac{TN}{TN + FP} \end{aligned}$$

Positive (*ppv*) and negative (*npv*) predictive values

The positive predictive values (*ppv*), or precision, measures the proportion of positive genomes present when a gene is present. Similarly, the negative predictive values (*npv*) measured the proportion of negative genomes are absent when a gene is absent.

$$\begin{aligned} ppv(g) &= P(\mathbf{G} \in \mathbf{E}_p^+ | g_i) = \frac{TP}{TP + FP} \\ npv(g) &= P(\mathbf{G} \in \mathbf{E}_p^- | \neg g_i) = \frac{TN}{TN + FN} \end{aligned}$$

Arithmetic (*amss*) and harmonic (*hmss*) means of sensitivity and specificity

Both scoring functions *amss* and *hmss* balance the rates of co-presence and co-absence. The *amss* scoring function is the arithmetic midpoint between sensitivity and specificity. The *hmss* scoring function, which defines the harmonic mean between the conditional probabilities, is conceptually similar to *amss* but it penalises genes with very low sensitivities or specificities.

$$\begin{aligned} amss(g) &= \frac{1}{2}\big(sens(g) + spec(g)\big) \\ hmss(g) &= \frac{1}{\frac{1}{sens(g)} + \frac{1}{spec(g)}} \end{aligned}$$

Odds ratios (*OR*)

The odds ratio compares the odds of a gene present in the positive example versus the odds of a gene absent in the negative examples, such that:

$$OR(g) = \frac{\frac{TP}{FP}}{\frac{FN}{TN}} = \frac{TP \times TN}{FP \times FN}$$

Chi-square (*chisq*) and directional chi-square (*bchisq*) scoring functions

χ^2 is a frequently-used statistic in testing variations between groups in discrete data. The *chisq* scoring function measured the deviation of the observed frequency from the expected proportion such that:

$$\begin{aligned} chisq(g) &= \sum_{i=1}^{n} \frac{(O_i - E_i)^2}{E_i} \\ &= \sum_{i=1}^{2}\sum_{j=1}^{2} \frac{\big(a_{ij} - E(a_{ij})\big)^2}{E(a_{ij})} \end{aligned}$$

where $E(a_{ij}) = \frac{(a_{1j}+a_{2j})(a_{i1}+a_{i2})}{a_{11}+a_{21}+a_{12}+a_{22}}$, a_{ij} = elements in the 2×2 contingency table.

The directional chi-square function (*bchisq*) is similar to *chisq*, but genes that display an inverse association are reversed to the bottom of the rank. *bchisq* excludes genes that are inversely associated with p.

$$bchisq(g) = \begin{cases} +chisq(g) & \text{if } OR(g) >= 1 \\ -chisq(g) & \text{if } OR(g) < 1 \end{cases}$$

F-measure (*F*)

F-measure is a frequently used statistic in evaluating performance of information retrieval systems. It is defined as the harmonic mean between the sensitivity and precision, such that:

$$F(g) = \frac{1}{\frac{1}{sens(g)} + \frac{1}{ppv(g)}}$$
