# Supplementary material for: In silico prioritisation of candidate genes for prokaryotic gene function discovery: an application of phylogenetic profiles
Source: BMC Bioinformatics. 2009 Mar 17;10:86. doi: 10.1186/1471-2105-10-86 (PMC2669486; doi:10.1186/1471-2105-10-86)
Supplement: Additional file 3 — The genes and the validation sets of peptidoglycan-related genes used in Case study 1. [file 1471-2105-10-86-S3.pdf]

Table A-3: Peptidoglycan-related genes

The loci and the validation sets of the peptidoglycan-related genes in Case study 1.

Gene	Gene product	Validation set	Genomes	
			SA-2603	EC-K12
Peptidoglycan biosynthesis and associated pathways				
*mur*A	UDP-*N*-acetylglucosamine 1-carboxyvinyltransferase	C, B, M	SAG0843<br>SAG0866	b3189
*mur*B	UDP-*N*-acetylmuramate dehydrogenase	C, B, M	SAG1112	b3972
*mur*C	UDP-*N*-acetylmuramate–alanine ligase	C, B, M	SAG1615	b0091
*mur*D	UDP-*N*-acetylmuramoylalanine–D-glutamate ligase	C, B, M	SAG0475	b0088
*mur*E	UDP-*N*-acetylmuramoylalanyl-D-glutamate–2,6-diaminopimelate ligase	C, B, M	SAG1391	b0085
*mur*F	UDP-*N*-acetylmuramoylalanyl-D-glutamyl-2,6-diaminopimelate–D-alanyl-D-alanine ligase	C, B, M	SAG0768	b0086
*mra*Y	phospho-*N*-acetylmuramoyl-pentapeptide-transferase	C, B, M	SAG0288	b0087
*mur*G	UDP-*N*-acetylglucosamine–*N*-acetylmuramyl-(pentapeptide)-pyrophosphoryl-undecaprenol N-acetylglucosamine transferase	C, B, M	SAG0476	b0090
UDP-*N*-acetylmuramate biosynthesis				
*glm*U	glucosamine-1-phosphate N-acetyltransferase UDP-*N*-acetylglucosamine pyrophosphorylase	B, M	SAG1538	b3730
*glm*M	phosphoglucomutase / phosphomannomutase	B, M	SAG0887	b3176
*glm*S	glucosamine–fructose-6-phosphate aminotransferase (isomerizing)	B, M	SAG0944	b3729
Undecarprenyl biosynthesis and recycling				
*upp*P/*bac*A	undecaprenyl pyrophosphate phosphatase	B, M	SAG0138	b3057

(Continue on next page)

Table 1: Peptidoglycan-related genes (continued)

Gene	Gene product	Validation set	Genomes	
			SA-2603	EC-K12
*upp*S/*isp*U	undecaprenyl diphosphate synthase	B, M	SAG1916	b0174
D-alanyl-D-alanine metabolism				
ddl	D-alanine-D-alanine ligase	B, M	SAG0767	b0381
				b0092
alr/*dad*X	alanine racemase	B, M	SAG1684	b1190
				b1190
D-glutamate metabolism				
*mur*I	glutamate racemase	B, M	SAG1600	b3967
meso-diaminopimelate biosynthesis				
*dap*F	diaminopimelate epimerase	B, M		b3809
*dap*E	N-succinyl-diaminopimelate deacylase	B, M		b2472
*dap*C	acetylornithine delta-aminotransferase	B, M		b3359
*dap*D	2,3,4,5-tetrahydropyridine-2-carboxylate N-succinyltransferase	B, M		b0166
*dap*B	dihydrodipicolinate reductase	B, M		b0031
*dap*A	dihydrodipicolinate synthase	B, M		b2478
asd	aspartate-semialdehyde dehydrogenase	B, M		b3433
*lys*C	aspartokinase	B, M		b4024
Peptidoglycan modification				
ami	*N*-acetylmuramoyl-L-alanine amidase	M	SAG0094	

(Continue on next page)

Table 1: Peptidoglycan-related genes (continued)

Gene	Gene product	Validation set	Genomes	
			SA-2603	EC-K12
*ami*A	*N*-acetylmuramoyl-L-alanine amidase	M		b2435
*ami*B	*N*-acetylmuramoyl-L-alanine amidase	M		b4169
*ami*C	*N*-acetylmuramoyl-L-alanine amidase	M		b2817
*ybj*R	*N*-acetylmuramoyl-L-alanine amidase	M		b0867
*amp*D	*N*-acetyl-anhydromuranmyl-L-alanine amidase	M		b0110
*amp*G	muropeptide transporter	M		b0433
*mlt*A	membrane-bound lytic murein transglycosylase A	M		b2813
*mlt*B	membrane-bound lytic murein transglycosylase B	M		b2701
*mlt*C	membrane-bound lytic murein transglycosylase C	M		b2963
*mlt*D	membrane-bound lytic murein transglycosylase D (predicted)	M		b0211
slt	lytic murein transglycosylase, soluble	M		b4392
*mep*A	murein DD-endopeptidase	M		b2328
*gln*A	glutamine synthetase	M	SAG1763	b3870
mpl	UDP-*N*-acetylmuramate:L-alanyl-gamma-D-glutamyl- meso-diaminopimelate ligase	M		b4233
Penicillin-binding proteins				
*pbp*1A	penicillin-binding protein 1A	M	SAG0298	b3396
*pbp*1B	penicillin-binding protein 1B	M	SAG0159	
*pbp*2/*mrd*A	penicillin-binding protein 2	M		b0635
*pbp*2A	pencillin binding protein 2A	M	SAG2066	
*pbp*2B	pencillin binding protein 2B	M	SAG0765	
*pbp*2X	pencillin binding protein 2X	M	SAG0287	

(Continue on next page)

Table 1: Peptidoglycan-related genes (continued)

Gene	Gene product	Validation set	Genomes	
			SA-2603	EC-K12
*pbp*3/*pbp*B	pencillin binding protein 3	M		b0084
*fts*I	carboxy-terminal protease for penicillin-binding protein 3 (EC-K12)	M		b1830
*mrc*B	fused glycosyl transferase and transpeptidase	M		b0149
*pbp*4	penicillin-binding protein 4	M	SAG0146	
*pbp*5/*dac*A	penicillin-binding protein 5: D-alanyl-D-alanine carboxypeptidase	M		b0632
*dac*C	penicillin-binding protein 6a/b: D-alanyl-D-alanine carboxypeptidase	M		b0839
*dac*D	penicillin-binding protein 6a/b: D-alanyl-D-alanine carboxypeptidase	M		b2010
*pbp*G	D-alanyl-D-alanine endopeptidase	M		b2134
*pbp*C	transglycosylase/transpeptidase	M		b2519
*mtg*A	biosynthetic peptidoglycan transglycosylase	M		b3208

(End of table)
