# Supplementary material for: In silico prioritisation of candidate genes for prokaryotic gene function discovery: an application of phylogenetic profiles
Source: BMC Bioinformatics. 2009 Mar 17;10:86. doi: 10.1186/1471-2105-10-86 (PMC2669486; doi:10.1186/1471-2105-10-86)
Supplement: Additional file 4 — This file lists the 400 positive and 17 negative genome examples used in statistical CGP of peptidoglycan-related genes. [file 1471-2105-10-86-S4.pdf]

Table A-4: Positive and negative genome examples used in the statistical CGP of peptidoglycan-related genes (case study 1)

This table lists the 400 positive genome examples and 17 negative genome examples in the statistical CGP of peptidoglycan-related genes.

Genome	Genbank Accession
Positive genome examples (400)	
Acidobacteria bacterium Ellin345	CP000360
Acidothermus cellulolyticus 11B	CP000481
Acidovorax JS42	CP000539, CP000540, CP000541
Acidovorax avenae citrulli AAC00-1	CP000512
Acinetobacter sp ADP1	CR543861
Aeromonas hydrophila ATCC 7966	CP000462
Agrobacterium tumefaciens C58 UWash	AE008687, AE008688, AE008689, AE008690
Alcanivorax borkumensis SK2	AM286690
Alkalilimnicola ehrlichei MLHE-1	CP000453
Anabaena variabilis ATCC 29413	CP000117, CP000119, CP000120, CP000121
Anaeromyxobacter dehalogenans 2CP-C	CP000251
Aquifex aeolicus	AE000657, AE000667
Arthrobacter FB24	CP000454
Arthrobacter aurescens TC1	CP000474, CP000475, CP000476
Azoarcus BH72	AM406670
Azoarcus sp EbN1	CR555306, CR555307, CR555308
Bacillus anthracis Ames	AE016879
Bacillus anthracis Ames 0581	AE017334, AE017335, AE017336
Bacillus anthracis str Sterne	AE017225
Bacillus cereus ATCC14579	AE016877, AE016878
Bacillus cereus ATCC 10987	AE017194, AE017195

(Continue on next page)

Genome	Genbank Accession
Bacillus cereus ZK	CP000001, CP000040, CP000041, CP000042, CP000043, CP000044
Bacillus clausii KSM-K16	AP006627
Bacillus halodurans	BA000004
Bacillus licheniformis DSM 13	AE017333
Bacillus subtilis	AL009126
Bacillus thuringiensis Al Hakam	CP000485, CP000486
Bacillus thuringiensis konkukian	AE017355, CP000047
Bacteroides fragilis NCTC 9434	CR626927, CR626928
Bacteroides fragilis YCH46	AP006841, AP006842
Bacteroides thetaiotaomicron VPI-5482	AE015928, AY171301
Bartonella bacilliformis KC583	CP000524
Bartonella henselae Houston-1	BX897699
Bartonella quintana Toulouse	BX897700
Baumannia cicadellinicola Homalodisca coagulata	CP000238
Bdellovibrio bacteriovorus	BX842601
Bifidobacterium adolescentis ATCC 15703	AP009256
Bifidobacterium longum	AE014295, AF540971
Bordetella bronchiseptica	BX470250
Bordetella parapertussis	BX470249
Bordetella pertussis	BX470248
Borrelia afzelii PKo	CP000395, CP000396, CP000397, CP000398, CP000399, CP000400, CP000401, CP000402, CP000403

(Continue on next page)

Genome	Genbank Accession
Borrelia burgdorferi	AE000784, AE000785, AE000786, AE000787, AE000788, AE000789, AE000790, AE000791, AE000792, AE000793, AE000794, AE001115, AE001575, AE001576, AE001577, AE001578, AE001579, AE001580, AE001581, AE001582, AE001583, AE001584
Borrelia garinii PBi	CP000013, CP000014, CP000015
Bradyrhizobium japonicum	BA000040
Brucella abortus 9-941	AE017223, AE017224
Brucella melitensis	AE008917, AE008918
Brucella melitensis biovar Abortus	AM040264, AM040265
Brucella suis 1330	AE014291, AE014292
Buchnera aphidicola	AE016826, AF492591
Buchnera aphidicola Cc Cinara cedri	CP000263
Buchnera aphidicola Sg	AE013218
Buchnera sp	AP001070, AP001071, BA000003
Burkholderia 383	CP000150, CP000151, CP000152
Burkholderia cenocepacia AU 1054	CP000378, CP000379, CP000380

(Continue on next page)

Genome	Genbank Accession
Burkholderia cenocepacia HI2424	CP000458,
	CP000459,
	CP000460,
	CP000461
Burkholderia cepacia AMMD	CP000440,
	CP000441,
	CP000442,
	CP000443
Burkholderia mallei ATCC 23344	CP000010,
	CP000011
Burkholderia mallei NCTC 10229	CP000545,
	CP000546
Burkholderia mallei SAVP1	CP000525,
	CP000526
Burkholderia pseudomallei 1710b	CP000124,
	CP000125
Burkholderia pseudomallei K96243	BX571965,
	BX571966
Burkholderia thailandensis E264	CP000085,
	CP000086
Burkholderia xenovorans LB400	CP000270,
	CP000271,
	CP000272
Campylobacter fetus 82-40	CP000487
Campylobacter jejuni	AL111168
Campylobacter jejuni RM1221	CP000025
Candidatus Blochmannia floridanus	BX248583
Candidatus Blochmannia pennsylvanicus BPEN	CP000016
Candidatus Carsonella ruddii	AP009180
Candidatus Pelagibacter ubique HTCC1062	CP000084
Candidatus Ruthia magnifica Cm Calyptogena magnifica	CP000488
Carboxydothermus hydrogenoformans Z-2901	CP000141
Caulobacter crescentus	AE005673
Chlamydia muridarum	AE002160,
	AE002162
Chlamydia trachomatis	AE001273
Chlamydia trachomatis A HAR-13	CP000051,
	CP000052
Chlamydophila abortus S26 3	CR848038
Chlamydophila caviae	AE015925,
	AE015926
Chlamydophila felis Fe C-56	AP006861,
	AP006862
Chlamydophila pneumoniae AR39	AE002161
Chlamydophila pneumoniae CWL029	AE001363
Chlamydophila pneumoniae J138	BA000008
Chlamydophila pneumoniae TW 183	AE009440

(Continue on next page)

Genome	Genbank Accession
Chlorobium chlorochromatii CaD3	CP000108
Chlorobium phaeobacteroides DSM 266	CP000492
Chlorobium tepidum TLS	AE006470
Chromobacterium violaceum	AE016825
Chromohalobacter salexigens DSM 3043	CP000285
Clostridium acetobutylicum	AE001437,<br>AE001438
Clostridium novyi NT	CP000382
Clostridium perfringens	AP003515,<br>BA000016
Clostridium perfringens ATCC 13124	CP000246
Clostridium perfringens SM101	CP000312,<br>CP000313,<br>CP000314,<br>CP000315
Clostridium tetani E88	AE015927,<br>AF528097
Clostridium thermocellum ATCC 27405	
Colwellia psychrerythraea 34H	CP000083
Corynebacterium diphtheriae	BX248353
Corynebacterium efficiens YS-314	BA000035
Corynebacterium glutamicum ATCC 13032 Bielefeld	BX927147
Corynebacterium jeikeium K411	AF401314,<br>CR931997
Coxiella burnetii	AE016828,<br>AE016829
Cyanobacteria bacterium Yellowstone A-Prime	CP000239
Cyanobacteria bacterium Yellowstone B-Prime	CP000240
Cytophaga hutchinsonii ATCC 33406	CP000383
Dechloromonas aromatica RCB	CP000089
Dehalococcoides CBDB1	AJ965256
Dehalococcoides ethenogenes 195	CP000027
Deinococcus geothermalis DSM 11300	CP000358,<br>CP000359
Deinococcus radiodurans	AE000513,<br>AE001825,<br>AE001826,<br>AE001827
Desulfitobacterium hafniense Y51	AP008230
Desulfotalea psychrophila LSv54	CR522870,<br>CR522871,<br>CR522872
Desulfovibrio desulfuricans G20	CP000112
Desulfovibrio vulgaris DP4	CP000527,<br>CP000528
Desulfovibrio vulgaris Hildenborough	AE017285,<br>AE017286

(Continue on next page)

Genome	Genbank Accession
Ehrlichia canis Jake	CP000107
Ehrlichia chaffeensis Arkansas	CP000236
Ehrlichia ruminantium Gardel	CR925677
Ehrlichia ruminantium str. Welgevonden	CR925678
Enterococcus faecalis V583	AE016830, AE016831, AE016832, AE016833
Erwinia carotovora atroseptica SCRI1043	BX950851
Erythrobacter litoralis HTCC2594	CP000157
Escherichia coli 536	CP000247
Escherichia coli APEC O1	CP000468
Escherichia coli CFT073	AE014075
Escherichia coli K12	U00096
Escherichia coli O157H7	AB011548, AB011549, BA000007
Escherichia coli O157H7 EDL933	AE005174, AF074613
Escherichia coli UTI89	CP000243, CP000244
Escherichia coli W3110	
Francisella tularensis FSC 198	AM286280
Francisella tularensis holarctica	AM233362
Francisella tularensis holarctica OSU18	CP000437
Francisella tularensis novicida U112	CP000439
Francisella tularensis tularensis	AJ749949
Frankia CcI3	CP000249
Frankia alni ACN14a	CT573213
Fusobacterium nucleatum	AE009951
Geobacillus kaustophilus HTA426	AP006520, BA000043
Geobacter metallireducens GS-15	CP000148, CP000149
Geobacter sulfurreducens	AE017180
Gloeobacter violaceus	BA000045
Gluconobacter oxydans 621H	CP000004, CP000005, CP000006, CP000007, CP000008, CP000009
Gramella forsetii KT0803	CU207366
Granulobacter bethesdensis CGDNIH1	CP000394
Haemophilus ducreyi 35000HP	AE017143
Haemophilus influenzae	
Haemophilus influenzae 86 028NP	CP000057

(Continue on next page)

Genome	Genbank Accession
Haemophilus somnus 129PT	CP000019, CP000436
Hahella chejuensis KCTC 2396	CP000155
Halorhodospira halophila SL1	CP000544
Helicobacter acinonychis Sheeba	AM260522, AM260523
Helicobacter hepaticus	AE017125
Helicobacter pylori 26695	AE000511
Helicobacter pylori HPAG1	CP000241, CP000242
Helicobacter pylori J99	AE001439
Hyphomonas neptunium ATCC 15444	CP000158
Idiomarina loihiensis L2TR	AE017340
Jannaschia CCS1	CP000264, CP000265
Lactobacillus acidophilus NCFM	CP000033
Lactobacillus brevis ATCC 367	CP000416, CP000417, CP000418
Lactobacillus casei ATCC 334	CP000423, CP000424
Lactobacillus delbrueckii bulgaricus	CR954253
Lactobacillus delbrueckii bulgaricus ATCC BAA-365	CP000412
Lactobacillus gasseri ATCC 33323	CP000413
Lactobacillus johnsonii NCC 533	AE017198
Lactobacillus plantarum	AL935263, CR377164, CR377165, CR377166
Lactobacillus sakei 23K	CR936503
Lactobacillus salivarius UCC118	AF488831, AF488832, CP000233, CP000234
Lactococcus lactis	AE005176
Lactococcus lactis cremoris MG1363	AM406671
Lactococcus lactis cremoris SK11	CP000425, CP000426, CP000427, CP000428, CP000429, CP000430
Lawsonia intracellularis PHE MN1-00	AM180252, AM180253, AM180254, AM180255

(Continue on next page)

Genome	Genbank Accession
Legionella pneumophila Lens	CR628337, CR628339
Legionella pneumophila Paris	CR628336, CR628338
Legionella pneumophila Philadelphia 1	AE017354
Leifsonia xyli xyli CTCB0	AE016822
Leptospira borgpetersenii serovar Hardjo-bovis JB197	CP000350, CP000351
Leptospira borgpetersenii serovar Hardjo-bovis L550	CP000348, CP000349
Leptospira interrogans serovar Copenhageni	AE016823, AE016824
Leptospira interrogans serovar Lai	AE010300, AE010301
Leuconostoc mesenteroides ATCC 8293	CP000414, CP000415
Listeria innocua	AL592022, AL592102
Listeria monocytogenes	AL591824
Listeria monocytogenes 4b F2365	AE017262
Listeria welshimeri serovar 6b SLCC5334	AM263198
Magnetococcus MC-1	CP000471
Magnetospirillum magneticum AMB-1	AP007255
Mannheimia succiniciproducens MBEL55E	AE016827
Maricaulis maris MCS10	CP000449
Marinobacter aquaeolei VT8	
Mesorhizobium BNC1	CP000389, CP000390, CP000391, CP000392
Mesorhizobium loti	AP003017, BA000012, BA000013
Methylibium petroleiphilum PM1	CP000555, CP000556
Methylobacillus flagellatus KT	CP000284
Methylococcus capsulatus Bath	AE017282
Moorella thermoacetica ATCC 39073	CP000232
Mycobacterium KMS	CP000518, CP000519, CP000520
Mycobacterium MCS	CP000384, CP000385
Mycobacterium avium 104	CP000479
Mycobacterium avium paratuberculosis	AE016958
Mycobacterium bovis	BX248333
Mycobacterium bovis BCG Pasteur 1173P2	AM408590

(Continue on next page)

Genome	Genbank Accession
Mycobacterium leprae	AL450380
Mycobacterium smegmatis MC2 155	CP000480
Mycobacterium tuberculosis CDC1551	AE000516
Mycobacterium tuberculosis H37Rv	AL123456
Mycobacterium ulcerans Agy99	CP000325
Mycobacterium vanbaalenii PYR-1	CP000511
Myxococcus xanthus DK 1622	CP000113
Neisseria gonorrhoeae FA 1090	AE004969
Neisseria meningitidis FAM18	AM421808
Neisseria meningitidis MC58	AE002098
Neisseria meningitidis Z2491	AL157959
Neorickettsia sennetsu Miyayama	CP000237
Nitrobacter hamburgensis X14	CP000319, CP000320, CP000321, CP000322
Nitrobacter winogradskyi Nb-255	CP000115
Nitrosococcus oceani ATCC 19707	CP000126, CP000127
Nitrosomonas europaea	AL954747
Nitrosomonas eutropha C71	CP000450, CP000451, CP000452
Nitrosospira multiformis ATCC 25196	CP000103, CP000104, CP000105, CP000106
Nocardia farcinica IFM10152	AP006618, AP006619, AP006620
Nocardioides JS614	CP000508, CP000509
Nostoc sp	AP003602, AP003603, AP003604, AP003605, AP003606, BA000019, BA000020
Novosphingobium aromaticivorans DSM 12444	CP000248
Oceanobacillus iheyensis	BA000028
Oenococcus oeni PSU-1	CP000411
Parachlamydia sp UWE25	BX908798
Paracoccus denitrificans PD1222	CP000489, CP000490, CP000491
Pasteurella multocida	AE004439

(Continue on next page)

Genome	Genbank Accession
Pediococcus pentosaceus ATCC 25745	CP000422
Pelobacter carbinolicus	CP000142
Pelobacter propionicus DSM 2379	CP000482, CP000483, CP000484
Pelodictyon luteolum DSM 273	CP000096
Photobacterium profundum SS9	CR354531, CR354532, CR377818
Photorhabdus luminescens	BX470251
Pirellula sp	BX119912
Polaromonas JS666	CP000316, CP000317, CP000318
Polaromonas naphthalenivorans CJ2	CP000529, CP000530, CP000531, CP000532, CP000533, CP000534, CP000535, CP000536, CP000537
Porphyromonas gingivalis W83	AE015924
Prochlorococcus marinus AS9601	CP000551
Prochlorococcus marinus CCMP1375	AE017126
Prochlorococcus marinus MED4	BX548174
Prochlorococcus marinus MIT9313	BX548175
Prochlorococcus marinus MIT 9303	CP000554
Prochlorococcus marinus MIT 9312	CP000111
Prochlorococcus marinus MIT 9515	CP000552
Prochlorococcus marinus NATL1A	CP000553
Prochlorococcus marinus NATL2A	CP000095
Propionibacterium acnes KPA171202	AE017283
Pseudoalteromonas atlantica T6c	CP000388
Pseudoalteromonas haloplanktis TAC125	CR954246, CR954247
Pseudomonas aeruginosa	AE004091
Pseudomonas aeruginosa UCBPP-PA14	CP000438
Pseudomonas entomophila L48	CT573326
Pseudomonas fluorescens Pf-5	CP000076
Pseudomonas fluorescens PfO-1	CP000094
Pseudomonas putida KT2440	AE015451
Pseudomonas syringae phaseolicola 1448A	CP000058, CP000059, CP000060
Pseudomonas syringae pv B728a	CP000075

(Continue on next page)

Genome	Genbank Accession
Pseudomonas syringae tomato DC3000	AE016853, AE016854, AE016855
Psychrobacter arcticum 273-4	CP000082
Psychrobacter cryohalolentis K5	CP000323, CP000324
Psychromonas ingrahamii 37	CP000510
Ralstonia eutropha H16	AM260479, AM260480
Ralstonia eutropha JMP134	CP000090, CP000091, CP000092, CP000093
Ralstonia metallidurans CH34	CP000352, CP000353, CP000354, CP000355
Ralstonia solanacearum	AL646052, AL646053
Rhizobium etli CFN 42	CP000133, CP000134, CP000135, CP000136, CP000137, CP000138
Rhizobium leguminosarum bv viciae 3841	AM236080, AM236081, AM236082, AM236083, AM236084, AM236085, AM236086
Rhodobacter sphaeroides 2 4 1	CP000143, CP000144, CP000145, CP000146, CP000147, DQ232586, DQ232587
Rhodococcus RHA1	CP000431, CP000432, CP000433, CP000434
Rhodoferax ferrireducens T118	CP000267, CP000268
Rhodopseudomonas palustris BisA53	CP000463
Rhodopseudomonas palustris BisB18	CP000301

(Continue on next page)

Genome	Genbank Accession
Rhodopseudomonas palustris BisB5	CP000283
Rhodopseudomonas palustris CGA009	BX571963, BX571964
Rhodopseudomonas palustris HaA2	CP000250
Rhodospirillum rubrum ATCC 11170	CP000230, CP000231
Rickettsia bellii RML369-C	CP000087
Rickettsia conorii	AE006914
Rickettsia felis URRWXCal2	CP000053, CP000054, CP000055
Rickettsia prowazekii	AJ235269
Rickettsia typhi wilmington	AE017197
Roseobacter denitrificans OCh 114	CP000362, CP000464, CP000465, CP000466, CP000467
Rubrobacter xylanophilus DSM 9941	CP000386
Saccharophagus degradans 2-40	CP000282
Salinibacter ruber DSM 13855	CP000159, CP000160
Salmonella enterica Choleraesuis	AE017220, AY509003, AY509004
Salmonella enterica Paratypi ATCC 9150	CP000026
Salmonella typhi	AL513382, AL513383, AL513384
Salmonella typhi Ty2	AE014613
Salmonella typhimurium LT2	AE006468, AE006471
Shewanella ANA-3	CP000469, CP000470
Shewanella MR-4	CP000446
Shewanella MR-7	CP000444, CP000445
Shewanella W3-18-1	CP000503
Shewanella amazonensis SB2B	CP000507
Shewanella denitrificans OS217	CP000302
Shewanella frigidimarina NCIMB 400	CP000447
Shewanella oneidensis	AE014299, AE014300
Shigella boydii Sb227	CP000036, CP000037
Shigella dysenteriae	CP000034, CP000035

(Continue on next page)

Genome	Genbank Accession
Shigella flexneri 2a	AE005674,<br>AF386526
Shigella flexneri 2a 2457T	AE014073
Shigella flexneri 5 8401	CP000266
Shigella sonnei Ss046	CP000038,<br>CP000039
Silicibacter TM1040	CP000375,<br>CP000376,<br>CP000377
Silicibacter pomeroyi DSS-3	CP000031,<br>CP000032
Sinorhizobium meliloti	AE006469,<br>AL591688,<br>AL591985
Sodalis glossinidius morsitans	AP008232,<br>AP008233,<br>AP008234,<br>AP008235
Solibacter usitatus Ellin6076	CP000473
Sphingopyxis alaskensis RB2256	CP000356,<br>CP000357
Staphylococcus aureus COL	CP000045,<br>CP000046
Staphylococcus aureus MW2	BA000033
Staphylococcus aureus Mu50	AP003367,<br>BA000017
Staphylococcus aureus N315	AP003139,<br>BA000018
Staphylococcus aureus NCTC 8325	CP000253
Staphylococcus aureus RF122	AJ938182
Staphylococcus aureus USA300	CP000255,<br>CP000256,<br>CP000257,<br>CP000258
Staphylococcus aureus aureus MRSA252	BX571856
Staphylococcus aureus aureus MSSA476	BX571857,<br>BX571858
Staphylococcus epidermidis ATCC 12228	AE015929,<br>AE015930,<br>AE015931,<br>AE015932,<br>AE015933,<br>AE015934,<br>AE015935
Staphylococcus epidermidis RP62A	CP000028,<br>CP000029
Staphylococcus haemolyticus	AP006716

(Continue on next page)

Genome	Genbank Accession
Staphylococcus saprophyticus	AP008934, AP008935, AP008936
Streptococcus agalactiae 2603	AE009948
Streptococcus agalactiae A909	CP000114
Streptococcus agalactiae NEM316	AL732656
Streptococcus mutans	AE014133
Streptococcus pneumoniae D39	CP000410
Streptococcus pneumoniae R6	AE007317
Streptococcus pyogenes M1 GAS	AE004092
Streptococcus pyogenes MGAS10270	CP000260
Streptococcus pyogenes MGAS10394	CP000003
Streptococcus pyogenes MGAS10750	CP000262
Streptococcus pyogenes MGAS2096	CP000261
Streptococcus pyogenes MGAS315	AE014074
Streptococcus pyogenes MGAS5005	CP000017
Streptococcus pyogenes MGAS6180	CP000056
Streptococcus pyogenes MGAS8232	AE009949
Streptococcus pyogenes MGAS9429	CP000259
Streptococcus pyogenes SSI-1	BA000034
Streptococcus sanguinis SK36	
Streptococcus thermophilus CNRZ1066	CP000024
Streptococcus thermophilus LMD-9	CP000419, CP000420, CP000421
Streptococcus thermophilus LMG 18311	CP000023
Streptomyces avermitilis	AP005645, BA000030
Streptomyces coelicolor	AL589148, AL645771, AL645882
Symbiobacterium thermophilum IAM14863	AP006840
Synechococcus CC9311	CP000435
Synechococcus CC9605	CP000110
Synechococcus CC9902	CP000097
Synechococcus elongatus PCC 6301	AP008231
Synechococcus elongatus PCC 7942	CP000100, CP000101
Synechococcus sp WH8102	BX548020
Synechocystis PCC6803	AP004310, AP004311, AP004312, AP006585, BA000022
Syntrophobacter fumaroxidans MPOB	CP000478
Syntrophomonas wolfei Goettingen	CP000448
Syntrophus aciditrophicus SB	CP000252

(Continue on next page)

Genome	Genbank Accession
Thermoanaerobacter tengcongensis	AE008691
Thermobifida fusca YX	CP000088
Thermosynechococcus elongatus	BA000039
Thermotoga maritima	AE000512
Thermus thermophilus HB27	AE017221, AE017222
Thermus thermophilus HB8	AP008226, AP008227, AP008228
Thiobacillus denitrificans ATCC 25259	CP000116
Thiomicrospira crunogena XCL-2	CP000109
Thiomicrospira denitrificans ATCC 33889	CP000153
Treponema denticola ATCC 35405	AE017226
Treponema pallidum	AE000520
Trichodesmium erythraeum IMS101	CP000393
Tropheryma whipplei TW08 27	BX072543
Tropheryma whipplei Twist	AE014184
Verminephrobacter eiseniae EF01-2	CP000542, CP000543
Vibrio cholerae	AE003852, AE003853
Vibrio fischeri ES114	CP000020, CP000021, CP000022
Vibrio parahaemolyticus	BA000031, BA000032
Vibrio vulnificus CMCP6	AE016795, AE016796
Vibrio vulnificus YJ016	AP005352, BA000037, BA000038
Wigglesworthia brevipalpis	AB063523, BA000021
Wolbachia endosymbiont of Brugia malayi TRS	AE017321
Wolbachia endosymbiont of Drosophila melanogaster	AE017196
Wolinella succinogenes	BX571656
Xanthomonas campestris	AE008922
Xanthomonas campestris 8004	CP000050
Xanthomonas campestris vesicatoria 85-10	AM039948, AM039949, AM039950, AM039951, AM039952
Xanthomonas citri	AE008923, AE008924, AE008925
Xanthomonas oryzae KACC10331	

(Continue on next page)

Genome	Genbank Accession
Xanthomonas oryzae MAFF 311018	AP008229
Xylella fastidiosa	AE003849, AE003850, AE003851
Xylella fastidiosa Temecula1	AE009442, AE009443
Yersinia enterocolitica 8081	
Yersinia pestis Antiqua	CP000308, CP000309, CP000310, CP000311
Yersinia pestis CO92	AL109969, AL117189, AL117211, AL590842
Yersinia pestis KIM	AE009952, AF074611
Yersinia pestis Nepal516	CP000305, CP000306, CP000307
Yersinia pestis biovar Mediaevails	AE017042, AE017043, AE017044, AE017045, AE017046
Yersinia pseudotuberculosis IP32953	BX936398, BX936399, BX936400
Zymomonas mobilis ZM4	AE008692
Negative genome examples (17)	
Anaplasma marginale St Maries	CP000030
Anaplasma phagocytophilum HZ	CP000235
Aster yellows witches-broom phytoplasma AYWB	CP000061, CP000062, CP000063, CP000064, CP000065
Mesoplasma florum L1	AE017263
Mycoplasma capricolum ATCC 27343	CP000123
Mycoplasma gallisepticum	AE015450
Mycoplasma hyopneumoniae 232	AE017332
Mycoplasma hyopneumoniae 7448	AE017244
Mycoplasma hyopneumoniae J	AE017243
Mycoplasma mobile 163K	AE017308
Mycoplasma mycoides	BX293980
Mycoplasma penetrans	BA000026
Mycoplasma pneumoniae	U00089

(Continue on next page)

Genome	Genbank Accession
Mycoplasma pulmonis	AL445566
Mycoplasma synoviae 53	AE017245
Onion yellows phytoplasma	AP006628
Ureaplasma urealyticum	AF222894

(End of table)
