# Supplementary material for: In silico prioritisation of candidate genes for prokaryotic gene function discovery: an application of phylogenetic profiles
Source: BMC Bioinformatics. 2009 Mar 17;10:86. doi: 10.1186/1471-2105-10-86 (PMC2669486; doi:10.1186/1471-2105-10-86)
Supplement: Additional file 5 — This file lists the positions of glycolysis genes in the ranks produced by statistical CGP of peptidoglycan genes. [file 1471-2105-10-86-S5.pdf]

Table A-5: Genes and the ranks of control validation set (glycolysis) used in Case study 1

Gene	Gene product	SA-2603		EC-K12	
		Locus	*pct*	Locus	*pct*
glk	glucokinase	SAG0471	19.5	b2388	36.4
pgi	glucose-6-phosphate isomerase	SAG0402	95.8	b4025	5.9
*pfk*A	6-phosphofructokinase	SAG0940	97.6	b1723	49.7
				b3916	99.6
fba, *dhn*A	fructose-bisphosphate aldolase	SAG0127	97.7	b2097	96.1
				b2925	99.7
gap	glyceraldehyde-3-phosphate dehydrogenase	SAG1768	94.0	b1779	3.0
pgk	phosphoglycerate kinase	SAG1766	94.5	b2926	2.4
gpm	phosphoglycerate mutase family protein	SAG0092	31.6	b0755	7.2
		SAG0752	16.9	b3612	100.0
		SAG0764	5.0	b4395	17.0
eno	phosphopyruvate hydratase	SAG0628	32.1	b2779	11.3
pyk	pyruvate kinase	SAG0941	55.7	b1676	83.9
				b1854	6.8
*ycc*X	acylphosphatase	SAG1607	38.4	b0968	9.5

Table 1: Case study 1: the position (in *pct*) of glycolysis genes using *amss* scoring functions on peptidoglycan genome examples
