# Supplementary material for: In silico prioritisation of candidate genes for prokaryotic gene function discovery: an application of phylogenetic profiles
Source: BMC Bioinformatics. 2009 Mar 17;10:86. doi: 10.1186/1471-2105-10-86 (PMC2669486; doi:10.1186/1471-2105-10-86)
Supplement: Additional file 7 — The rank positions, rank fractions (in pct), cluster of orthologous groups (COG), and the positions of candidate genes in the reference genome (SA-2603) ranked by amss scoring function. [file 1471-2105-10-86-S7.pdf]

Table A-7: Prioritised rank (*amss* scoring function) on SA-2603 genes

The rank position, rank fraction (in *pct*), cluster of orthologous groups (COG), and loci of the prioritised rank by *amss* scoring function in the SA-2603 genome.

Rank	*pct*	Score	COG	Valid. Set	Gene	Locus	Function
1	0.05	0.97	M	M	*pbp*2A	SAG2066	penicillin-binding protein 2A
2	0.09	0.97	O		*gro*ES	SAG2075	co-chaperonin GroES
3	0.14	0.96			*aro*C	SAG1377	chorismate synthase
3	0.14	0.96	M	B,M	*alr*	SAG1684	alanine racemase
5	0.24	0.96	M	B,M	*ddl*	SAG0767	D-alanylalanine synthetase
6	0.28	0.96	M	M	*pbp*1A	SAG0298	penicillin-binding protein 1A
7	0.33	0.96	M	M		SAG0159	penicillin-binding protein 1B, putative
7	0.33	0.96	E		*aro*B	SAG1378	3-dehydroquinate synthase
9	0.42	0.96	M	C,B,M	*mur*D	SAG0475	UDP-*N*-acetylmuramoyl-L-alanyl-D-glutamate synthetase
9	0.42	0.96			*fts*W	SAG0761	cell division protein, FtsW/RodA/SpoVE family
9	0.42	0.96	M	C,B,M	*mur*F	SAG0768	UDP-*N*-acetylmuramoylalanyl-D-glutamyl-2,6-diaminopimelate–D-alanyl-D-alanyl ligase
9	0.42	0.96	M	C,B,M	*mur*A-1	SAG0843	UDP-*N*-acetylglucosamine 1-carboxyvinyltransferase
9	0.42	0.96	M	C,B,M	*mur*A-2	SAG0866	UDP-*N*-acetylglucosamine 1-carboxyvinyltransferase
14	0.66	0.96	M		*pbp*X	SAG0287	penicillin-binding protein 2X
14	0.66	0.96	M	B,M	*mra*Y	SAG0288	phospho-*N*-acetylmuramoyl-pentapeptide-transferase
14	0.66	0.96	M	C,B,M	*mur*C	SAG1615	UDP-*N*-acetylmuramate–L-alanine ligase
17	0.8	0.95	M			SAG0140	glycosyl transferase, group 4 family protein
18	0.85	0.95	E		*aro*E	SAG1680	shikimate 5-dehydrogenase
19	0.89	0.95	TK			SAG0322	DNA-binding response regulator
20	0.94	0.95	E		*cys*K	SAG0334	cysteine synthase A
21	0.99	0.95	M	B,M	*mur*E	SAG1391	UDP-*N*-acetylmuramoylalanyl-D-glutamate–2,6-diaminopimelate ligase
22	1.04	0.95	T			SAG1507	phoH family protein

(Continue on next page)

Rank	*pct*	Score	COG	Valid. Set	Gene	Locus	Function
23	1.08	0.94	M	B,M	*glm*U	SAG1538	UDP-*N*-acetylglucosamine pyrophosphorylase
24	1.13	0.94	R			SAG0480	ylmE protein, putative
25	1.18	0.94	O		*rad*A	SAG0110	DNA repair protein RadA
26	1.22	0.94	Q		*dlt*A	SAG1790	D-alanine–D-alanyl carrier protein ligase
27	1.27	0.94			*rod*A	SAG0621	rod shape-determining protein RodA, putative
28	1.32	0.94	EH			SAG1528	chorismate binding enzyme
29	1.37	0.94	O		*clp*X	SAG1312	ATP-dependent protease ATP-binding subunit
29	1.37	0.94	OU		*clp*P	SAG1585	ATP-dependent Clp protease proteolytic subunit
31	1.46	0.94	J		*rps*A	SAG1150	30S ribosomal protein S1
32	1.51	0.94	H			SAG0498	geranyltranstransferase, putative
32	1.51	0.94	H			SAG1738	polyprenyl synthetase family protein
34	1.6	0.94	E			SAG0339	aspartate kinase
35	1.65	0.94	O			SAG1530	peptidyl-prolyl cis-trans isomerase, cyclophilin-type
36	1.69	0.94	I		*acc*C	SAG0352	acetyl-CoA carboxylase
37	1.74	0.93	M	B,M	*mur*I	SAG1600	glutamate racemase
38	1.79	0.93	L		*nth*	SAG0468	endonuclease III
							⋮
Remaining peptidoglycan genes							
43	2.02	0.93	I	B,M	*upp*S	SAG1916	undecaprenyl diphosphate synthase
56	2.64	0.92	M	B,M	*glm*S	SAG0944	D-fructose-6-phosphate amidotransferase
62	2.92	0.92		B,M	*upp*P	SAG0138	undecaprenyl pyrophosphate phosphatase
73	3.44	0.92	M	C,B,M	*mur*G	SAG0476	*N*-acetylglucosaminyl transferase
90	4.24	0.91	M	M		SAG0765	penicillin-binding protein 2b
108	5.08	0.90		B,M	*gln*A	SAG1763	glutamine synthetase, type I
159	7.49	0.86	M	C,B,M	*mur*B	SAG1112	UDP-*N*-acetylenolpyruvoylglucosamine reductase
264	12.43	0.81	G	B,M		SAG0887	phosphoglucomutase/phosphomannomutase family protein
276	12.99	0.81	M	M		SAG0146	penicillin-binding protein 4, putative
566	26.65	0.66	NU	M		SAG0094	*N*-acetylmuramoyl-L-alanine amidase, family 4 protein

(End of table)
