# Supplementary material for: In silico prioritisation of candidate genes for prokaryotic gene function discovery: an application of phylogenetic profiles
Source: BMC Bioinformatics. 2009 Mar 17;10:86. doi: 10.1186/1471-2105-10-86 (PMC2669486; doi:10.1186/1471-2105-10-86)
Supplement: Additional file 9 — The rank fraction (in pct) of genes prioritised by amss scoring function in the EC-K12 genome. [file 1471-2105-10-86-S9.pdf]

## Table A-9: Anaerobic mixed-acid fermenation genes ranked by *amss* scoring function

The rank fraction (in *pct*) and the gene loci of the prioritised rank by *amss* scoring function in the EC-K12 genome.

| Gene | Gene product | Locus | *pct* |
|---|---|---|---|
| Genes specific to mixed-acid fermentation | | | |
| Pyruvate formate-lyase complex | | | |
| *pfl*A | pyruvate formate-lyase activating enzyme [E.C. 1.97.1.4] | b0902 | 0.19 |
| *pfl*B | pyruvate formate lyase I [E.C. 2.3.1.54] | b0903 | 0.15 |
| *pfl*D | formate C-acetyltransferase [E.C. 2.3.1.54] | b3951 | 0.17 |
| *tdc*E | pyruvate formate-lyase 4/2-ketobutyrate formate-lyase | b3114 | 0.05 |
| *pfl*C | pyruvate formate lyase II activase | b3952 | 0.07 |
| *ybi*Y | predicted pyruvate formate lyase activating enzyme | b0824 | 0.07 |
| *yjj*W | predicted pyruvate formate lyase activating enzyme | b4379 | 0.07 |
| *ybi*W | predicted pyruvate formate lyase | b0823 | 0.22 |
| *yfi*D | pyruvate formate lyase subunit | b2579 | 0.27 |
| Acetate formation | | | |
| *pta* | phosphate acetyltransferase [E.C. 2.3.1.8] | b2297 | 12.03 |
| *ack*A | acetate kinase [E.C. 3.6.1.7] | b2296 | 2.08 |
| Ethanol and lactate formation | | | |
| *adh*E | alcohol / acetaldehyde dehydrogenase [E.C. 1.1.1.1 and 1.2.1.10] | b1241 | 2.06 |
| *ldh*A | D-lactate dehydrogenase (NAD dependent) [E.C. 1.1.1.28] | b1380 | 66.28 |
| Formate hydrogenlyase complex | | | |
| *hyc*A | regulator of the transcriptional regulator FhlA | b2725 | 30.19 |
| *hyc*B | hydrogenase 3, Fe-S subunit | b2724 | 10.34 |
| *hyc*C | hydrogenase 3, membrane subunit | b2723 | 99.27 |
| *hyc*D | hydrogenase 3, membrane subunit | b2722 | 99.71 |
| *hyc*E | hydrogenase 3, large subunit | b2721 | 99.83 |
| *hyc*F | formate hydrogenlyase complex iron-sulfur protein | b2720 | 99.69 |
| *hyc*G | hydrogenase 3 and formate hydrogenase complex, HycG subunit | b2719 | 99.93 |

*(Continue on next page)*

| Gene | Gene product | Locus | *pct* |
|---|---|---|---|
| *hyc*H | protein required for maturation of hydrogenase 3 | b2718 | 21.98 |
| *hyc*I | protease involved in processing C-terminal end of HycE | b2717 | 23.00 |
| *fdh*D | formate dehydrogenase formation protein | b3895 | 70.61 |
| *fdh*E | formate dehydrogenase formation protein | b3891 | 15.47 |
| *fdh*F | formate dehydrogenase-H, selenopolypeptide subunit | b4079 | 88.31 |
| Fumarate/Succinate formation | | | |
| *frd*A | fumarate reductase (anaerobic) catalytic and NAD/flavoprotein subunit | b4154 | 93.00 |
| *frd*B | fumarate reductase (anaerobic), Fe-S subunit | b4153 | 99.15 |
| *frd*C | fumarate reductase (anaerobic), membrane anchor subunit | b4152 | 10.38 |
| *frd*D | fumarate reductase (anaerobic), membrane anchor subunit | b4151 | 7.84 |
| Genes that also share with other pathways | | | |
| Pyruvate kinases | | | |
| *pyk*F | pyruvate kinase I | b1676 | 41.06 |
| *pyk*A | pyruvate kinase II | b1854 | 41.06 |
| *ppc* | phosphoenolpyruvate carboxylase | b3956 | 57.15 |
| Fumarate/Succinate formation | | | |
| *mdh* | malate dehydrogenase, NAD(P)-binding | b3236 | 2.35 |
| *fum*A | fumarate hydratase (fumarase A), aerobic Class I | b1612 | 61.49 |
| *fum*B | anaerobic class I fumarate hydratase (fumarase B) | b4122 | 61.49 |
| *fum*C | fumarate hydratase (fumarase C),aerobic Class II | b1611 | 95.59 |
| Ethanol and Lactate formation | | | |
| *lld*D | FMN- linked L-lactate dehydrogenase | b3605 | 91.77 |
| *adh*P | ethanol-active dehydrogenase/acetaldehyde-active reductase | b1478 | 87.9 |

*(End of table)*
