# Supplementary material for: In silico prioritisation of candidate genes for prokaryotic gene function discovery: an application of phylogenetic profiles
Source: BMC Bioinformatics. 2009 Mar 17;10:86. doi: 10.1186/1471-2105-10-86 (PMC2669486; doi:10.1186/1471-2105-10-86)
Supplement: Additional file 10 — The rank positions, rank fractions (in pct), cluster of orthologous groups (COG), and the positions of candidate genes in the reference genome (E. coli K-12) prioritised by amss scoring function. [file 1471-2105-10-86-S10.pdf]

Table A-10: Gene rank produced by amss scoring function (anaerobic mixed-acid fermentation)

The rank position, rank fraction (in *pct*), cluster of orthologous groups (COG), and the loci of the prioritised rank by *amss* scoring function in the EC-K12 genome.

Rank	*pct*	Score	COG	Gene	Locus	Function
1	0.02	0.806	F	*nrd*D	b4238	anaerobic ribonucleoside-triphosphate reductase
2	0.05	0.806	C	*tdc*E	b3114	pyruvate formate-lyase 4/2-ketobutyrate formate-lyase
3	0.07	0.805	O	*ybi*Y	b0824	predicted pyruvate formate lyase activating enzyme
3	0.07	0.805	O	*pfl*C	b3952	pyruvate formate lyase II activase
3	0.07	0.805	O	*yjj*W	b4379	predicted pyruvate formate lyase activating enzyme
6	0.15	0.803	C	*pfl*B	b0903	pyruvate formate lyase I
7	0.17	0.802	C	*pfl*D	b3951	predicted formate acetyltransferase 2 (pyruvate formate lyase II)
8	0.19	0.797	O	*pfl*A	b0902	pyruvate formate lyase activating enzyme 1
9	0.22	0.787	C	*ybi*W	b0823	predicted pyruvate formate lyase
10	0.24	0.781	O	*nrd*G	b4237	anaerobic ribonucleotide reductase activating protein
11	0.27	0.781	R	*yfi*D	b2579	pyruvate formate lyase subunit
12	0.29	0.774	R	*yhc*C	b3211	predicted Fe-S oxidoreductase
13	0.31	0.765	R	*ybh*A	b0766	predicted hydrolase
14	0.34	0.757	E	*pep*T	b1127	peptidase T
15	0.36	0.748	G	*nag*E	b0679	fused *N*-acetyl glucosamine specific PTS enzyme: IIC, IIB , and IIA components
15	0.36	0.748		*crr*	b2417	glucose-specific enzyme IIA component of PTS
15	0.36	0.748	G	*bgl*F	b3722	fused beta-glucoside-specific PTS enzymes: IIA component/IIB component/IIC component
18	0.44	0.746	G	*tre*B	b4240	fused trehalose(maltose)-specific PTS enzyme: IIB component/IIC component
19	0.46	0.744	G	*mur*P	b2429	fused predicted PTS enzymes: IIB component/IIC component
20	0.48	0.737	G	*asc*F	b2715	fused cellobiose/arbutin/salicin-specific PTS enzymes: IIB component/IC component

(Continue on next page)

Rank	*pct*	Score	COG	Gene	Locus	Function
21	0.51	0.734	P	*thi*I	b0423	sulfurtransferase required for thiamine and 4-thiouridine biosynthesis
22	0.53	0.733	G	*pts*H	b2415	phosphohistidinoprotein-hexose phosphotransferase component of PTS system (Hpr)
23	0.56	0.732	E	*brn*Q	b0401	predicted branched chain amino acid transporter (LIV-II)
24	0.58	0.725	F	*deo*D	b4384	purine-nucleoside phosphorylase
25	0.61	0.721	R	*ybi*V	b0822	predicted hydrolase
26	0.63	0.721	G	*nag*B	b0678	glucosamine-6-phosphate deaminase
27	0.65	0.718	P	*nir*C	b3367	nitrite transporter
28	0.68	0.715	G	*chb*C	b1737	*N*,*N*'-diacetylchitobiose-specific enzyme IIC component of PTS
29	0.70	0.714		*man*Z	b1819	mannose-specific enzyme IID component of PTS
29	0.70	0.714		*aga*V	b3133	N-acetylgalactosamine-specific enzyme IIB component of PTS
31	0.75	0.713	R	*cof*	b0446	thiamin pyrimidine pyrophosphate hydrolase
32	0.77	0.711	S	*yjj*P	b4364	predicted inner membrane protein
33	0.80	0.710	P	*foc*B	b2492	predicted formate transporter
34	0.82	0.710		*lux*S	b2687	S-ribosylhomocysteinase
35	0.85	0.709		*aga*D	b3140	*N*-acetylgalactosamine-specific enzyme IID component of PTS
36	0.87	0.708	G	*aga*I	b3141	galactosamine-6-phosphate isomerase
37	0.90	0.707		*man*Y	b1818	mannose-specific enzyme IIC component of PTS
38	0.92	0.707	GT	*ula*C	b4195	L-ascorbate-specific enzyme IIA component of PTS
39	0.94	0.707		*ula*A	b4193	L-ascorbate-specific enzyme IIC component of PTS
40	0.97	0.706		*aga*B	b3138	*N*-acetylgalactosamine-specific enzyme IIB component of PTS

(End of table)
