# Supplementary material for: In silico prioritisation of candidate genes for prokaryotic gene function discovery: an application of phylogenetic profiles
Source: BMC Bioinformatics. 2009 Mar 17;10:86. doi: 10.1186/1471-2105-10-86 (PMC2669486; doi:10.1186/1471-2105-10-86)
Supplement: Additional file 11 — This is the tabular representation of results in Figure5, showing the AUCs of 10-fold cross-validations in rediscovering genes in the 31 KEGG pathways evaluated in Case study 3. [file 1471-2105-10-86-S11.pdf]

Table A-11: Stratified cross-validation results of inductive CGP algorithms on 31 selected KEGG pathways

Table 1: Case study 3: cross-validation results of inductive CGP algorithms on 31 selected KEGG pathways

KEGG pathway or functional group	Algorithms						
	NB	*LR*	*ADTree*	*IBk*	*J48*	*SVM/P*	*SVM/R*
ABC transporters - General	0.754	0.861	0.873	**0.914**	0.824	0.912	0.905
Alanine and aspartate metabolism	0.802	0.737	0.770	0.737	0.785	0.795	**0.809**
Aminoacyl-tRNA biosynthesis	0.886	0.928	**0.960**	0.905	0.926	0.929	0.893
Aminosugars metabolism	0.725	0.674	**0.923**	0.827	0.639	0.899	0.762
Arginine and proline metabolism	0.766	**0.962**	0.907	0.875	0.821	0.935	0.943
Butanoate metabolism	0.589	0.750	0.681	0.765	0.563	0.671	**0.784**
Carbon fixation	0.789	0.724	0.764	**0.799**	0.715	0.791	0.728
DNA replication	0.790	0.789	0.853	0.810	0.854	**0.860**	0.791
Fatty acid biosynthesis	0.893	0.989	**0.994**	0.974	0.776	0.988	0.857
Folate biosynthesis	0.751	0.787	0.849	**0.861**	0.607	0.836	0.812
Fructose and mannose metabolism	0.656	0.524	0.723	**0.795**	0.632	0.787	0.626
Galactose metabolism	0.643	0.742	0.791	0.811	0.743	**0.875**	0.831
Glutamate metabolism	0.766	0.767	0.810	**0.872**	0.746	0.802	0.797
Glycine and serine and threonine metabolism	0.746	0.772	**0.843**	0.755	0.730	0.802	0.812
Glycolysis / Gluconeogenesis	0.752	0.879	**0.908**	0.873	0.808	0.894	0.879
Methionine metabolism	0.739	0.846	0.812	0.753	0.777	**0.858**	0.793
Oxidative phosphorylation	0.666	0.740	0.785	**0.906**	0.720	0.806	0.751
Pentose and glucuronate interconversions	0.803	0.720	0.919	0.933	0.721	**0.939**	0.808
Pentose phosphate pathway	0.717	0.795	0.898	**0.948**	0.737	0.894	0.852
Peptidoglycan biosynthesis	0.760	0.882	0.853	0.902	0.653	0.757	**0.940**
Phenylalanine and tyrosine and tryptophan biosynthesis	0.709	0.663	0.668	0.715	0.621	0.682	**0.729**
Phosphotransferase system (PTS)	0.883	0.949	0.962	0.961	0.864	**0.990**	0.975
Propanoate metabolism	0.739	0.969	**0.985**	0.964	0.675	0.905	0.919
Protein export	0.646	0.788	0.745	0.692	0.646	**0.857**	0.749
Purine metabolism	0.740	0.776	0.789	**0.813**	0.783	0.797	0.791
Pyrimidine metabolism	0.761	0.666	**0.816**	0.741	0.678	0.713	0.718
Pyruvate metabolism	0.749	0.789	0.853	0.844	0.503	**0.868**	0.824
Starch and sucrose metabolism	0.792	0.738	0.766	**0.866**	0.746	0.858	0.772
Two-component system - General	0.783	0.698	0.772	0.772	0.723	**0.893**	0.840
Tyrosine metabolism	0.760	0.806	0.693	**0.936**	0.654	0.727	0.832
Ribosome	0.794	0.851	**0.930**	0.897	0.812	0.910	0.922
Number of pathways in which the algorithm achieved best AUC	0	1	8	10	0	8	4

Values shown in this table are the areas under ROC curves. The **bold** values indicated the best AUC was achieved by the algorithm in the pathway evaluated. *NB*: naïve Bayes classifier; *LR*: logistic regression; *ADTree*: alternating decision tree; *IBk*: k-nearest neighbour; *J48*: J48 decision tree; *SVM*: support vector machine; *SVM/P*: SVM with polynomial kernel; *SVM/R*: SVM with radial basis kernel.
