# Supplementary material for: In silico prioritisation of candidate genes for prokaryotic gene function discovery: an application of phylogenetic profiles
Source: BMC Bioinformatics. 2009 Mar 17;10:86. doi: 10.1186/1471-2105-10-86 (PMC2669486; doi:10.1186/1471-2105-10-86)
Supplement: Additional file 12 — In Case study 1, evaluation experiments were performed on candidate genes selected from one S. agalactiae and one E. coli genomes. These bacterial genomes belong to divisions of Firmicutes and Gamma-proteobacteria, both consisting of large number of closely-related sequences in positive examples, and it could have favourably biased the performance due to over-representation. This file describes an additional CGP experiment by selecting a less-well represented genome from the NCBI database, Prochlorococcus marinus MIT9313, to investigate this effect. [file 1471-2105-10-86-S12.pdf]

Additional file A-13 - CGP performance on peptidoglycan-related genes on *Prochlorococcus marinus* MIT9313

Movitation and Methods

In Case study 1, both statistical and inductive CGP methods were evaluated on genes of *Streptococcus agalactiae* 2603 V/R (SA-2603) and *Escherichia coli* K-12 (EC-K12) genomes with a large collection of genome examples incorporating the phyla of Firmicutes (99) and Proteobacteria (222). It is unclear whether the CGP results were favorably biased by the overrepresentation of these genome examples. To investigate the possibile bias, an additional experiment was designed to rank genes from the *Prochlorococcus marinus* MIT9313 genomes (PM-MIT9313, 2,269 genes) with all Cyanobacteria genomes removed from the 400 positive genomes examples (including all *Anabaena* spp., *Gloeobacter* spp., *Nostoc* spp., *Prochlorococcus* spp., *Synechococcus* spp., *Synechocystis* spp., *Thermosynechococcus* spp., and *Trichodesmium* spp.). The determination of occurrence matrix, statistical CGP scoring functions, inductive CGP algorithms, and evaluation methodology were identical to Case study 1.

Results

For statistical CGP, the best performing scoring function was *hmss* with high AUC (best AUC: C: 0.988, B: 0.971, M: 0.969). Good inductive CGP performance as measured by AUC were also obtained (C:0.999 by *ADTree*; B: 0.954 by *SVM/Poly*; M: 0.986 by *SVM/Poly*).

Discussion

This experiment attempted to prioritise PM-MIT9313 for discovering genes responsible for peptidoglycan metabolism *without* the representative genomes from the phylum Cyanobacteria. The high AUCs achieved by both methods were similar to the corresponding CGP tasks in SA-2603 and EC-K12 genomes. These results demonstrated that good CGP performances were obtainable by selecting positive genome examples from either closely- or distantly-related genomes.

Table 1: CGP performance on peptidoglycan-related genes (*Prochlorococcus marinus* MIT9313, 2269 genes)

Methods	Validation sets					
	C (8)		B (23)		M (30)	
	AUC	$(\overline{\eta}/\eta_{max})$	AUC	$(\overline{\eta}/\eta_{max})$	AUC	$(\overline{\eta}/\eta_{max})$
Statistical CGP (scoring functions)						
sens	0.887	(2.2/6.65)	0.867	(2.0/4.93)	0.858	(2.0/4.44)
spec	0.262	(0.3/1.33)	0.331	(0.5/1.18)	0.381	(0.7/1.18)
ppv	0.527	(0.8/1.96)	0.594	(1.4/24.7)	0.641	(1.5/37.8)
npv	0.970	(3.6/25.2)	0.953	(3.2/16.4)	0.946	(3.1/15.1)
amss	0.987	(4.6/75.6)	0.970	(4.0/41.5)	0.969	(4.0/43.8)
hmss	0.988	(4.8/87.3)	0.971	(4.0/45.5)	0.969	(4.0/47.3)
OR	0.521	(0.7/1.96)	0.593	(1.4/24.7)	0.638	(1.4/37.8)
chisq	0.980	(4.1/42.2)	0.963	(3.6/23.1)	0.957	(3.5/20.9)
bchisq	0.980	(4.1/42.2)	0.963	(3.6/23.1)	0.957	(3.5/20.9)
F	0.943	(3.3/29.9)	0.906	(2.8/16.4)	0.894	(2.7/15.1)
Inductive CGP (machine learning algorithms)						
NB	0.919		0.878		0.872	
LR	0.968		0.819		0.877	
ADTree	0.999		0.921		0.915	
IBk	0.833		0.952		0.960	
J48	0.993		0.818		0.705	
SMO/Poly	0.937		0.954		0.986	
SMO/RBF	0.995		0.898		0.855	

Genome examples selected for the statistical CGP experiment

Positive genome examples (378)

Acidobacteria bacterium Ellin345
Acidothermus cellulolyticus 11B
Acidovorax JS42
Acidovorax avenae citrulli AAC00-1
Acinetobacter sp ADP1
Aeromonas hydrophila ATCC 7966
Agrobacterium tumefaciens C58 UWash
Alcanivorax borkumensis SK2
Alkalilimnicola ehrlichei MLHE-1
Anabaena variabilis ATCC 29413
Anaeromyxobacter dehalogenans 2CP-C
Aquifex aeolicus
Arthrobacter FB24
Arthrobacter aurescens TC1
Azoarcus BH72
Azoarcus sp EbN1
Bacillus anthracis Ames
Bacillus anthracis Ames 0581
Bacillus anthracis str Sterne
Bacillus cereus ATCC14579
Bacillus cereus ATCC 10987
Bacillus cereus ZK
Bacillus clausii KSM-K16
Bacillus halodurans
Bacillus licheniformis DSM 13
Bacillus subtilis
Bacillus thuringiensis Al Hakam
Bacillus thuringiensis konkukian
Bacteroides fragilis NCTC 9434
Bacteroides fragilis YCH46
Bacteroides thetaiotaomicron VPI-5482
Bartonella bacilliformis KC583
Bartonella henselae Houston-1
Bartonella quintana Toulouse
Baumannia cicadellinicola Homalodisca coagulata
Bdellovibrio bacteriovorus
Bifidobacterium adolescentis ATCC 15703
Bifidobacterium longum
Bordetella bronchiseptica
Bordetella parapertussis
Bordetella pertussis
Borrelia afzelii PKo
Borrelia burgdorferi
Borrelia garinii PBi
Bradyrhizobium japonicum
Brucella abortus 9-941
Brucella melitensis
Brucella melitensis biovar Abortus
Brucella suis 1330
Buchnera aphidicola
Buchnera aphidicola Cc Cinara cedri
Buchnera aphidicola Sg
Buchnera sp
Burkholderia 383
Burkholderia cenocepacia AU 1054
Burkholderia cenocepacia HI2424
Burkholderia cepacia AMMD
Burkholderia mallei ATCC 23344
Burkholderia mallei NCTC 10229
Burkholderia mallei SAVP1
Burkholderia pseudomallei 1710b
Burkholderia pseudomallei K96243
Burkholderia thailandensis E264
Burkholderia xenovorans LB400
Campylobacter fetus 82-40
Campylobacter jejuni
Campylobacter jejuni RM1221
Candidatus Blochmannia floridanus
Candidatus Blochmannia pennsylvanicus BPEN
Candidatus Carsonella ruddii
Candidatus Pelagibacter ubique HTCC1062
Candidatus Ruthia magnifica Cm Calyptogena magnifica
Carboxydothermus hydrogenoformans Z-2901
Caulobacter crescentus
Chlamydia muridarum
Chlamydia trachomatis
Chlamydia trachomatis A HAR-13
Chlamydophila abortus S26 3

Chlamydophila caviae
Chlamydophila felis Fe C-56
Chlamydophila pneumoniae AR39
Chlamydophila pneumoniae CWL029
Chlamydophila pneumoniae J138
Chlamydophila pneumoniae TW 183
Chlorobium chlorochromatii CaD3
Chlorobium phaeobacteroides DSM 266
Chlorobium tepidum TLS
Chromobacterium violaceum
Chromohalobacter salexigens DSM 3043
Clostridium acetobutylicum
Clostridium novyi NT
Clostridium perfringens
Clostridium perfringens ATCC 13124
Clostridium perfringens SM101
Clostridium tetani E88
Clostridium thermocellum ATCC 27405
Colwellia psychrerythraea 34H
Corynebacterium diphtheriae
Corynebacterium efficiens YS-314
Corynebacterium glutamicum ATCC 13032 Bielefeld
Corynebacterium jeikeium K411
Coxiella burnetii
Cytophaga hutchinsonii ATCC 33406
Dechloromonas aromatica RCB
Dehalococcoides CBDB1
Dehalococcoides ethenogenes 195
Deinococcus geothermalis DSM 11300
Deinococcus radiodurans
Desulfitobacterium hafniense Y51
Desulfotalea psychrophila LSv54
Desulfovibrio desulfuricans G20
Desulfovibrio vulgaris DP4
Desulfovibrio vulgaris Hildenborough
Ehrlichia canis Jake
Ehrlichia chaffeensis Arkansas
Ehrlichia ruminantium Gardel
Ehrlichia ruminantium str. Welgevonden
Enterococcus faecalis V583
Erwinia carotovora atroseptica SCRI1043
Erythrobacter litoralis HTCC2594
Escherichia coli 536
Escherichia coli APEC O1
Escherichia coli CFT073
Escherichia coli K12
Escherichia coli O157H7
Escherichia coli O157H7 EDL933
Escherichia coli UTI89
Escherichia coli W3110
Francisella tularensis FSC 198
Francisella tularensis holarctica
Francisella tularensis holarctica OSU18
Francisella tularensis novicida U112
Francisella tularensis tularensis
Frankia CcI3
Frankia alni ACN14a
Fusobacterium nucleatum
Geobacillus kaustophilus HTA426
Geobacter metallireducens GS-15
Geobacter sulfurreducens
Gluconobacter oxydans 621H
Gramella forsetii KT0803
Granulobacter bethesdensis CGDNIH1
Haemophilus ducreyi 35000HP
Haemophilus influenzae
Haemophilus influenzae 86 028NP
Haemophilus somnus 129PT
Hahella chejuensis KCTC 2396
Halorhodospira halophila SL1
Helicobacter acinonychis Sheeba
Helicobacter hepaticus
Helicobacter pylori 26695
Helicobacter pylori HPAG1
Helicobacter pylori J99
Hyphomonas neptunium ATCC 15444
Idiomarina loihiensis L2TR
Jannaschia CCS1
Lactobacillus acidophilus NCFM
Lactobacillus brevis ATCC 367
Lactobacillus casei ATCC 334
Lactobacillus delbrueckii bulgaricus
Lactobacillus delbrueckii bulgaricus ATCC BAA-365
Lactobacillus gasseri ATCC 33323
Lactobacillus johnsonii NCC 533
Lactobacillus plantarum
Lactobacillus sakei 23K
Lactobacillus salivarius UCC118
Lactococcus lactis
Lactococcus lactis cremoris MG1363

Lactococcus lactis cremoris SK11
Lawsonia intracellularis PHE MN1-00
Legionella pneumophila Lens
Legionella pneumophila Paris
Legionella pneumophila Philadelphia 1
Leifsonia xyli xyli CTCB0
Leptospira borgpetersenii serovar Hardjobovis JB197
Leptospira borgpetersenii serovar Hardjobovis L550
Leptospira interrogans serovar Copenhageni
Leptospira interrogans serovar Lai
Leuconostoc mesenteroides ATCC 8293
Listeria innocua
Listeria monocytogenes
Listeria monocytogenes 4b F2365
Listeria welshimeri serovar 6b SLCC5334
Magnetococcus MC-1
Magnetospirillum magneticum AMB-1
Mannheimia succiniciproducens MBEL55E
Maricaulis maris MCS10
Marinobacter aquaeolei VT8
Mesorhizobium BNC1
Mesorhizobium loti
Methylibium petroleiphilum PM1
Methylobacillus flagellatus KT
Methylococcus capsulatus Bath
Moorella thermoacetica ATCC 39073
Mycobacterium KMS
Mycobacterium MCS
Mycobacterium avium 104
Mycobacterium avium paratuberculosis
Mycobacterium bovis
Mycobacterium bovis BCG Pasteur 1173P2
Mycobacterium leprae
Mycobacterium smegmatis MC2 155
Mycobacterium tuberculosis CDC1551
Mycobacterium tuberculosis H37Rv
Mycobacterium ulcerans Agy99
Mycobacterium vanbaalenii PYR-1
Myxococcus xanthus DK 1622
Neisseria gonorrhoeae FA 1090
Neisseria meningitidis FAM18
Neisseria meningitidis MC58
Neisseria meningitidis Z2491
Neorickettsia sennetsu Miyayama
Nitrobacter hamburgensis X14
Nitrobacter winogradskyi Nb-255
Nitrosococcus oceani ATCC 19707
Nitrosomonas europaea
Nitrosomonas eutropha C71
Nitrosospira multiformis ATCC 25196
Nocardia farcinica IFM10152
Nocardioides JS614
Novosphingobium aromaticivorans DSM 12444
Oceanobacillus iheyensis
Oenococcus oeni PSU-1
Parachlamydia sp UWE25
Paracoccus denitrificans PD1222
Pasteurella multocida
Pediococcus pentosaceus ATCC 25745
Pelobacter carbinolicus
Pelobacter propionicus DSM 2379
Pelodictyon luteolum DSM 273
Photobacterium profundum SS9
Photorhabdus luminescens
Pirellula sp
Polaromonas JS666
Polaromonas naphthalenivorans CJ2
Porphyromonas gingivalis W83
Propionibacterium acnes KPA171202
Pseudoalteromonas atlantica T6c
Pseudoalteromonas haloplanktis TAC125
Pseudomonas aeruginosa
Pseudomonas aeruginosa UCBPP-PA14
Pseudomonas entomophila L48
Pseudomonas fluorescens Pf-5
Pseudomonas fluorescens PfO-1
Pseudomonas putida KT2440
Pseudomonas syringae phaseolicola 1448A
Pseudomonas syringae pv B728a
Pseudomonas syringae tomato DC3000
Psychrobacter arcticum 273-4
Psychrobacter cryohalolentis K5
Psychromonas ingrahamii 37
Ralstonia eutropha H16
Ralstonia eutropha JMP134
Ralstonia metallidurans CH34
Ralstonia solanacearum
Rhizobium etli CFN 42
Rhizobium leguminosarum bv viciae 3841

Rhodobacter sphaeroides 2 4 1
Rhodococcus RHA1
Rhodoferax ferrireducens T118
Rhodopseudomonas palustris BisA53
Rhodopseudomonas palustris BisB18
Rhodopseudomonas palustris BisB5
Rhodopseudomonas palustris CGA009
Rhodopseudomonas palustris HaA2
Rhodospirillum rubrum ATCC 11170
Rickettsia bellii RML369-C
Rickettsia conorii
Rickettsia felis URRWXCal2
Rickettsia prowazekii
Rickettsia typhi wilmington
Roseobacter denitrificans OCh 114
Rubrobacter xylanophilus DSM 9941
Saccharophagus degradans 2-40
Salinibacter ruber DSM 13855
Salmonella enterica Choleraesuis
Salmonella enterica Paratypi ATCC 9150
Salmonella typhi
Salmonella typhi Ty2
Salmonella typhimurium LT2
Shewanella ANA-3
Shewanella MR-4
Shewanella MR-7
Shewanella W3-18-1
Shewanella amazonensis SB2B
Shewanella denitrificans OS217
Shewanella frigidimarina NCIMB 400
Shewanella oneidensis
Shigella boydii Sb227
Shigella dysenteriae
Shigella flexneri 2a
Shigella flexneri 2a 2457T
Shigella flexneri 5 8401
Shigella sonnei Ss046
Silicibacter TM1040
Silicibacter pomeroyi DSS-3
Sinorhizobium meliloti
Sodalis glossinidius morsitans
Solibacter usitatus Ellin6076
Sphingopyxis alaskensis RB2256
Staphylococcus aureus COL
Staphylococcus aureus MW2
Staphylococcus aureus Mu50
Staphylococcus aureus N315
Staphylococcus aureus NCTC 8325
Staphylococcus aureus RF122
Staphylococcus aureus USA300
Staphylococcus aureus aureus MRSA252
Staphylococcus aureus aureus MSSA476
Staphylococcus epidermidis ATCC 12228
Staphylococcus epidermidis RP62A
Staphylococcus haemolyticus
Staphylococcus saprophyticus
Streptococcus agalactiae 2603
Streptococcus agalactiae A909
Streptococcus agalactiae NEM316
Streptococcus mutans
Streptococcus pneumoniae D39
Streptococcus pneumoniae R6
Streptococcus pyogenes M1 GAS
Streptococcus pyogenes MGAS10270
Streptococcus pyogenes MGAS10394
Streptococcus pyogenes MGAS10750
Streptococcus pyogenes MGAS2096
Streptococcus pyogenes MGAS315
Streptococcus pyogenes MGAS5005
Streptococcus pyogenes MGAS6180
Streptococcus pyogenes MGAS8232
Streptococcus pyogenes MGAS9429
Streptococcus pyogenes SSI-1
Streptococcus sanguinis SK36
Streptococcus thermophilus CNRZ1066
Streptococcus thermophilus LMD-9
Streptococcus thermophilus LMG 18311
Streptomyces avermitilis
Streptomyces coelicolor
Symbiobacterium thermophilum IAM14863
Syntrophobacter fumaroxidans MPOB
Syntrophomonas wolfei Goettingen
Syntrophus aciditrophicus SB
Thermoanaerobacter tengcongensis
Thermobifida fusca YX
Thermotoga maritima
Thermus thermophilus HB27
Thermus thermophilus HB8
Thiobacillus denitrificans ATCC 25259
Thiomicrospira crunogena XCL-2
Thiomicrospira denitrificans ATCC 33889
Treponema denticola ATCC 35405

Treponema pallidum
Tropheryma whipplei TW08 27
Tropheryma whipplei Twist
Verminephrobacter eiseniae EF01-2
Vibrio cholerae
Vibrio fischeri ES114
Vibrio parahaemolyticus
Vibrio vulnificus CMCP6
Vibrio vulnificus YJ016
Wigglesworthia brevipalpis
Wolbachia endosymbiont of Brugia malayi TRS
Wolbachia endosymbiont of Drosophila melanogaster
Wolinella succinogenes
Xanthomonas campestris
Xanthomonas campestris 8004
Xanthomonas campestris vesicatoria 85-10
Xanthomonas citri
Xanthomonas oryzae KACC10331
Xanthomonas oryzae MAFF 311018
Xylella fastidiosa
Xylella fastidiosa Temecula1
Yersinia enterocolitica 8081
Yersinia pestis Antiqua
Yersinia pestis CO92
Yersinia pestis KIM
Yersinia pestis Nepal516
Yersinia pestis biovar Mediaevails
Yersinia pseudotuberculosis IP32953
Zymomonas mobilis ZM4

Negative genome examples (17)

Anaplasma marginale St Maries
Anaplasma phagocytophilum HZ
Aster yellows witches-broom phytoplasma AYWB
Mesoplasma florum L1
Mycoplasma capricolum ATCC 27343
Mycoplasma gallisepticum
Mycoplasma hyopneumoniae 232
Mycoplasma hyopneumoniae 7448
Mycoplasma hyopneumoniae J
Mycoplasma mobile 163K
Mycoplasma mycoides
Mycoplasma penetrans
Mycoplasma pneumoniae
Mycoplasma pulmonis
Mycoplasma synoviae 53
Onion yellows phytoplasma
Ureaplasma urealyticum
