# Supplementary material for: In silico prioritisation of candidate genes for prokaryotic gene function discovery: an application of phylogenetic profiles
Source: BMC Bioinformatics. 2009 Mar 17;10:86. doi: 10.1186/1471-2105-10-86 (PMC2669486; doi:10.1186/1471-2105-10-86)
Supplement: Additional file 13 — There were considerable variations in inductive CGP performance in Case study 3, and some variations is attributable to statistical uncertainties or algorithmic differences. The influence of pathway functions on CGP performance was, however, unclear. Nevertheless, it was observed that there may be limitations in using KEGG pathways as a validation source, where potential sampling biases could have explained a significant proportion of such variations. In this file, an additional experiment was performed to illustrate this effect. [file 1471-2105-10-86-S13.pdf]

Additional file 12 – Sampling bias with validation sets selected from KEGG

In case study 3, there were significant variations in inductive CGP performance between different KEGG pathways. Apart from true indiscriminability of functional categories by phylogenetic profiles, these variations could have been attributed to the *sampling bias* in the validation data sets. KEGG is an useful resource for illustrating the transformation of enzymatic substrates. To depict complex biochemical interactions, it is often required to include neighbouring metabolic pathways in the same pathway map. This may result in mixed gene occurrence profiles which can adversely influence inductive CGP performance.

We manually inspected the worst performing validation set (phenylalanine, tyrosine, and tryptophan biosynthesis pathway, KEGG pathway Id: sag00400), which has genes sharing with 7 (out 81) other functional categories of KEGG. A closer examination revealed that two tRNA synthases genes and alanine/aspartate aminotransferase genes were included in the validation sets. The repeated experiment with the removal of these genes resulted in a significantly improved overall performance (with best AUC of improved from 0.729 to 0.852, Table 1 and 2).

To investigate this effect further, we plotted the extent of gene sharing of different KEGG categories (Figure 1). It was observed that validation sets with few overlaps tend to have better performance (as shown in red, yellow, and blue groups). The validation sets with good AUC [for example, fatty acid biosynthesis (sag00061, AUC: 0.994) and ribosomal genes (sag03010, AUC: 0.930)] have relatively few connections, indicating that only the genes specific to a the function were included. More dense connections were found in pathways that achieved worse AUC (blue and grey groups); although some heavily-connected pathways (for example, the amino-tRNA biosynthesis pathway, sag00970, AUC:

Table 1: Comparison of AUC between the original and processed sag00400 validation sets

Dataset	Algorithm						
	NB	*LR*	*ADTree*	*IBk*	*J48*	*SVM/P*	*SVM/R*
Original (sag00400)	0.709	0.663	0.668	0.715	0.621	0.682	**0.729**
Processed (sag00400m)	0.684	0.652	0.664	0.773	0.669	**0.851**	0.759

The values shown in this table are areas under ROC curve. The generalisation performance was estimated by stratified cross-validation for each algorithm (10-fold cross-validation for sag00400, 9-fold for sag00400m).

Table 2: Genes in the original (sag00400) and the processed (sag00400m) phenylalanine, tyrosine and tryptophan biosynthesis KEGG pathway

Gene locus	Pathway		Gene	Annotation
	sag00400	sag00400m		
SAG0158	✓		*tyr*S	tyrosyl-tRNA synthetase
SAG0462	✓	✓	*trp*G	anthranilate synthase component II
SAG0525	✓		*asp*C	aspartate aminotransferase
SAG0540	✓	✓		hypothetical protein
SAG0630	✓	✓	*aro*A	3-phosphoshikimate 1-carboxyvinyltransferase
SAG0631	✓	✓	*aro*K	shikimate kinase
SAG0869	✓		*phe*S	phenylalanyl-tRNA synthetase subunit α
SAG0871	✓		*phe*T	phenylalanyl-tRNA synthetase subunit β
SAG1377	✓	✓	*aro*C	chorismate synthase
SAG1378	✓	✓	*aro*B	3-dehydroquinate synthase
SAG1379	✓	✓	*aro*D	3-dehydroquinate dehydratase
SAG1676	✓		*ala*T	aminotransferase AlaT
SAG1680	✓	✓	*aro*E	shikimate 5-dehydrogenase
SAG1686	✓	✓		phospho-2-dehydro-3-heoxyheptonate aldolase
n	14	9		

0.960) have distinct molecular functional properties (for example, synthesis amino-tRNAs). In the above example with the sag00400 validation set, removal of tRNA synthase and aminotransferase genes (sag00400m) resulted in a reduction of connections with neighbouring pathways. Such "purification" step led to an improvement in CGP performance.

Summary

KEGG is a standardised validation source that is frequently used for benchmarking *in silico* methods of gene function discovery. The map-style of gene curation in KEGG implies that a possibility of mixing functionally distinct genes in the same validation set. In the case of CGP by phylogenetic profiles, inclusion of genes from other functional groups may degrade the quality of training data and resulting in loss of performance. Evaluations of functional prediction methods must thus consider the effect of such sampling bias in the data set.

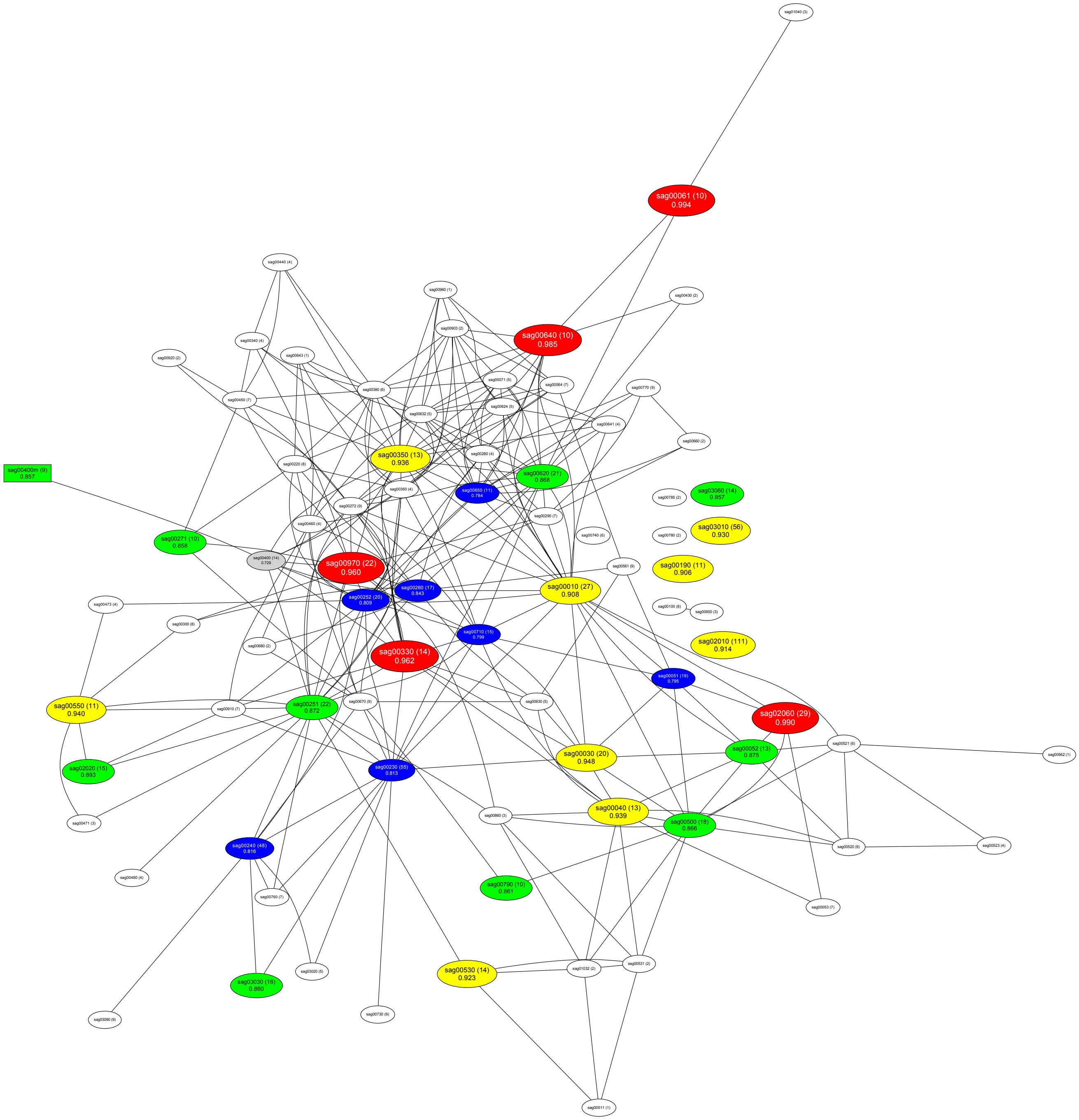

Figure 1: This diagram illustrates the pathways that have shared genes in KEGG. Each node denotes a KEGG functional category, and an edge between nodes indicates that common genes are present in both categories. Pathways with fewer connections are generally associated with better evaluation results, while pathways with poor AUCs are likely to be more heavily interconnected. The original validation set composed of phenylalanine, tyrosine, and tryptophan biosynthesis pathway in KEGG (sag00400, the grey node) contains genes sharing with more neighbouring pathways than the processed validation set (sag00400m, rectangular green node). The pathway names are listed in Table 3. Coloured nodes: red: AUC>0.95; yellow: AUC=0.90–0.95; green: AUC=0.85–0.90; blue: AUC=0.75–0.85; grey: AUC <0.75; White nodes: KEGG pathways with <10 genes in the pathway that (not included in inductive CGP evaluation).

Table 3: KEGG pathway listed in Figure 1

Id	Name
sag00010	Glycolysis / Gluconeogenesis
sag00030	Pentose phosphate pathway
sag00040	Pentose and glucuronate interconversions
sag00051	Fructose and mannose metabolism
sag00052	Galactose metabolism
sag00053	Ascorbate and aldarate metabolism
sag00061	Fatty acid biosynthesis
sag00071	Fatty acid metabolism
sag00100	Biosynthesis of steroids
sag00190	Oxidative phosphorylation
sag00220	Urea cycle and metabolism of amino groups
sag00230	Purine metabolism
sag00240	Pyrimidine metabolism
sag00251	Glutamate metabolism
sag00252	Alanine and aspartate metabolism
sag00260	Glycine, serine and threonine metabolism
sag00271	Methionine metabolism
sag00272	Cysteine metabolism
sag00280	Valine, leucine and isoleucine degradation
sag00290	Valine, leucine and isoleucine biosynthesis
sag00300	Lysine biosynthesis
sag00330	Arginine and proline metabolism
sag00340	Histidine metabolism
sag00350	Tyrosine metabolism
sag00360	Phenylalanine metabolism
sag00380	Tryptophan metabolism
sag00400	Phenylalanine, tyrosine and tryptophan biosynthesis
sag00430	Taurine and hypotaurine metabolism
sag00440	Aminophosphonate metabolism
sag00450	Selenoamino acid metabolism
sag00460	Cyanoamino acid metabolism
sag00471	D-Glutamine and D-glutamate metabolism
sag00473	D-Alanine metabolism
sag00480	Glutathione metabolism
sag00500	Starch and sucrose metabolism
sag00511	N-Glycan degradation
sag00520	Nucleotide sugars metabolism
sag00521	Streptomycin biosynthesis
sag00523	Polyketide sugar unit biosynthesis
sag00530	Aminosugars metabolism
sag00531	Glycosaminoglycan degradation

(Continue on next page)

Id	Name
sag00550	Peptidoglycan biosynthesis
sag00561	Glycerolipid metabolism
sag00562	Inositol phosphate metabolism
sag00564	Glycerophospholipid metabolism
sag00620	Pyruvate metabolism
sag00624	1- and 2-Methylnaphthalene degradation
sag00630	Glyoxylate and dicarboxylate metabolism
sag00632	Benzoate degradation via CoA ligation
sag00640	Propanoate metabolism
sag00641	3-Chloroacrylic acid degradation
sag00643	Styrene degradation
sag00650	Butanoate metabolism
sag00660	C5-Branched dibasic acid metabolism
sag00670	One carbon pool by folate
sag00680	Methane metabolism
sag00710	Carbon fixation
sag00730	Thiamine metabolism
sag00740	Riboflavin metabolism
sag00760	Nicotinate and nicotinamide metabolism
sag00770	Pantothenate and CoA biosynthesis
sag00780	Biotin metabolism
sag00785	Lipoic acid metabolism
sag00790	Folate biosynthesis
sag00860	Porphyrin and chlorophyll metabolism
sag00900	Terpenoid biosynthesis
sag00903	Limonene and pinene degradation
sag00910	Nitrogen metabolism
sag00920	Sulfur metabolism
sag00960	Alkaloid biosynthesis II
sag00970	Aminoacyl-tRNA biosynthesis
sag01032	Glycan structures - degradation
sag01040	Polyunsaturated fatty acid biosynthesis
sag02010	ABC transporters - General
sag02020	Two-component system - General
sag02060	Phosphotransferase system (PTS)
sag03010	Ribosome
sag03020	RNA polymerase
sag03030	DNA replication
sag03060	Protein export
sag03090	Type II secretion system

(End of table)
